# Recognizing distance-count matrices

**DOI:** 10.1371/journal.pone.0352427

**Published:** 2026-07-08

**Authors:** Paolo Boldi, Chiara Prezioso, Flavio Furia, Ian Stewart

**Affiliations:** 1 Computer Science Department, Università degli Studi, Milano, Italy; 2 Mathematics Institute, University of Warwick, Coventry, United Kingdom; Universidade de Sao Paulo, BRAZIL

## Abstract

Axiomatizing centrality measures often requires proving that certain properties do not hold by exhibiting a counterexample (i.e., a graph for which a given centrality measure does not satisfy a specified property). In the context of geometric centralities, constructing such counterexamples requires building a graph with prescribed distance counts, as encoded in its distance-count matrix (DCM). We prove that deciding whether a matrix is the distance-count matrix of an undirected graph is strongly NP-complete. This negative result implies that a brute-force approach to constructing such counterexamples is out of the question. We complement this negative result with some positive findings: while recognizing DCM matrices is strongly NP-hard, the construction of DCM matrices is algorithmically well-behaved under some natural graph operations (which we call *DCM-stable*): that is, for many important graph operations ⊗, the DCM of G⊗H can be computed efficiently from those of G and H, *without* having to reconstruct the graphs themselves. This observation shows that, although the inverse problem is intractable in general, distance-count matrices admit a rich and tractable compositional theory on structured graph classes generated by DCM-stable operations.

## 1 Introduction

The distance-count matrix (DCM) of a graph is a matrix whose rows correspond to the vertices, and where the *k*-th column for vertex *v* contains the number of vertices whose (shortest-path) distance to *v* is equal to *k*. The DCM contains a lot of information about the graph itself, and it is a natural object to study, especially in the context of social network analysis: a large family of centrality measures, called geometric centralities [1], are those that can be expressed as a function of the DCM of a graph. This family includes degree centrality, closeness [2], Lin centrality [3], harmonic centrality [1], and many others. As an example, the harmonic centrality of a node *i* in the graph G is defined as follows:


$$\begin{array}{c} {ℭ}_{G}^{harm}(i)=\sum_{j\in N_{G}}\frac{1}{d_{G}(j,i)}. \end{array}$$

where $N_{G}$ is the set of vertices of G, and $d_{G}(j,i)$ is the length of a shortest path from *j* to *i* in G; we understand $\frac{1}{d_{G}(j,i)}$ to be 0 if $d_{G}(j,i)$ is infinite (i.e., if there is no path from *j* to *i*). The intuition, here, is that a node is more central if it is closer to all other nodes in the graph; more central nodes have smaller distances, hence larger inverse-distance summation. To compute the harmonic centrality of a node *i*, we need to know how many nodes are at distance 1 to *i*, how many are at distance 2, and so on: this information is precisely encoded in the DCM of the graph. More precisely, if $N_{G}(i,t)$ is the number of nodes at distance *t* to *i* in G, and $|N_{G}|=n$, then we can rewrite the harmonic centrality of *i* as


$$\begin{array}{c} {ℭ}_{G}^{harm}(i)=\sum_{t=1}^{n-1}\frac{1}{t}\cdot N_{G}(i,t). \end{array}$$

The subfamily of linear geometric centralities alone was also studied in the pioneering works in Kishi *et al.* [4,5], and more recently in [6,7]. The DCM implicitly contains other information, such as the eccentricity of all vertices (and their distribution), the diameter and effective diameter [8] of the graph, the distance distribution [9], and the graph Wiener index [10,11]. The problem of computing or approximating the DCM is therefore extremely important in practice and challenging, especially for large graphs [12]. While building the DCM of a given graph is relatively easy, the main result we prove is that deciding whether a matrix is a DCM of an undirected graph is strongly NP-complete. This negative result is especially relevant when trying to build counterexamples to specific properties of geometric centralities: in such a situation, it may be possible to find a candidate DCM that works as a counterexample, but then the problem is to determine whether that candidate matrix *is* the actual DCM of some graph. Our result implies that this road is basically impossible, so we must explore a different technique for finding a counterexample.

We contrast this negative result with some positive observations. Even if recognizing whether a matrix is a DCM is extremely difficult, the construction of DCM matrices is algorithmically well-behaved under some natural graph operations. Let us call a graph operation ⊗ DCM-stable if the DCM of G⊗H can be computed efficiently from those of G and H, *without* having to reconstruct the graphs themselves: if this property holds, we call ⊗ DCM-stable.

We shall be able to prove that many common graph operations are DCM-stable. This observation shows that, although the inverse problem is intractable in general, distance-count matrices admit a rich and tractable compositional theory on structured graph classes generated by DCM-stable operations. Furthermore, DCM-stability has a concrete algorithmic payoff: one can get the DCM of exponentially large graphs (e.g., powers with respect to stable operations) without ever materializing the full graph. In other words, DCM matrices turn out to have a rich algebraic structure that mirrors the algebraic operations between the graphs they represent.

## 2 Related work

The rows of the distance-count matrices have been considered in the graph-theoretical literature under the name of “distance degree sequences” (or dds) [13, Chapter 5], and were studied in some special scenarios (e.g., to determine which graphs have vertices with pairwise distinct dds, and which have the same dds for all vertices [14]), but not in the general case.

Determining whether a given sequence of integers is a graphical degree sequence, that is, the degree sequence of an undirected graph, is a well-known problem in graph theory: among the first results about this problem are the celebrated Erdős-Gallai theorem [15], characterizing graphical degree sequences, and the Havel-Hakimi algorithm [16,17], which gives a constructive way to check in polynomial time whether a sequence is a graphical degree sequence, and provides, in the positive case, a possible realization of the sequence as a graph. Later, the problem was also studied in the context of directed graphs [18], where instead of the degree sequence one considers the in-degree and out-degree sequences.

Another quite natural generalization of the problem is to take a list of pairs of integers $\left(d_{i},d_{i}^{\prime }\right)$ and determine whether this is a second-order degree sequence of a simple undirected graph. Such a sequence contains, for every vertex *i*, the number of vertices $d_{i}$ at distance 1 (i.e., its degree) and the number of vertices $d_{i}^{\prime }$ at distance 2. This version of the problem, however, is strongly NP-complete [19]. In this paper, we consider an even more general version of the problem, where our input is the whole distance-count matrix of a graph.

The intuition that recognizing DCMs may itself be NP-complete comes from the fact that the first two columns of the DCM of a graph are precisely its second-order degree sequence: in the light of [19] we can expect that the problem of recognizing DCMs is at least as hard. But the DCM contains much more information than the second-order degree sequence: it contains the number of vertices at distance *k* for every *k*, and therefore it is a complete description of the distances in the graph. So, we may think that this additional knowledge could make the problem easier. Formally, there is no easy polynomial reduction between the two problems, in either direction.

## 3 Notation

A *graph* $G=(N_{G},E_{G})$ is given [20] by a finite set of nodes $N_{G}$ and a set of arcs $E_{G}\subseteq N_{G}\times N_{G}$; we assume that our graphs have no self loops, i.e., $(i,i)\notin E_{G}$ for all $i\in N_{G}$. (For this and similar notations, the subscript G is dropped whenever it is clear from the context.) Without loss of generality we let N=[n]={0,1,…,n−1} where *n* is the number of nodes. We write x→y to mean that x,y∈N and (x,y)∈E; we say that *x* is a *predecessor* of *y*, that *y* is a *successor* of *x*, and that *x* (*y*, respectively) is the *tail* (*head*, respectively) of the arc x→y. A graph is *undirected* iff x→y implies y→x. For undirected graphs, we write simply x↔y, and say that *x* and *y* are *adjacent*.

A *path* of length *k* from *x* to *y* is a sequence $\pi =(x_{0},x_{1},\ldots ,x_{k})$ of nodes such that *x* = *x*_0_, $y=x_{k}$ and $x_{i}\to x_{i+1}$ for all i∈[k]; the arcs $x_{i}\to x_{i+1}$ are said to *belong* to the path. We say that a path is *simple* if it does not contain any node twice.

**Fig 1:**
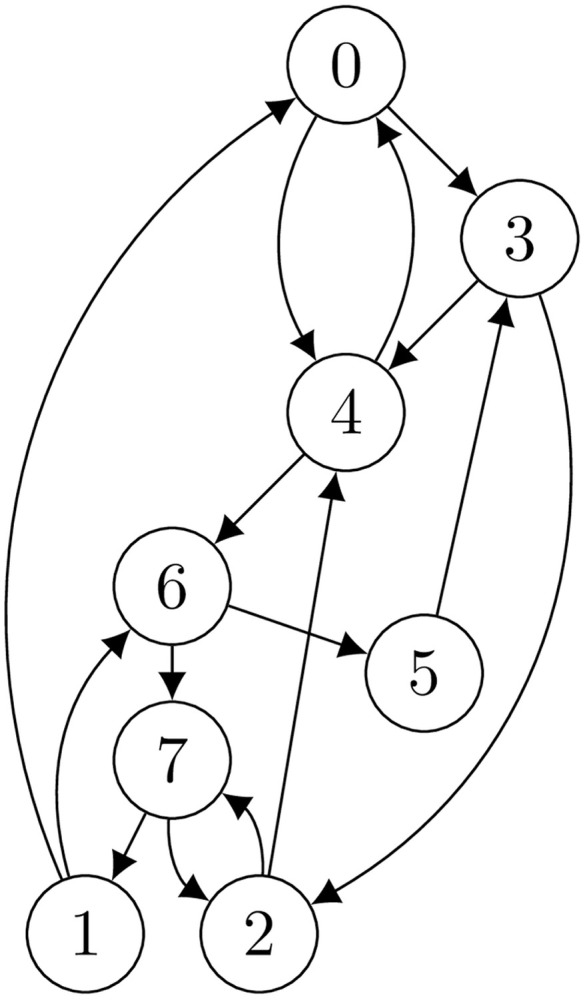
A strongly connected directed graph G.

The *(shortest path) distance* from *x* to *y* in G, denoted by $d_{G}(x,y)$, is the length of a shortest path from *x* to *y*, or ∞ if no path from *x* to *y* exists. A *(strongly) connected graph* is one where the distance between every pair of nodes is finite (the adverb “strongly” is usually omitted for undirected graphs). See, for instance, the graph of Fig 1.

In a connected undirected graph, the distance function d(−,−) is a metric, that is, for all x,y,z∈N (1) d(x,y)≥0 and equality holds if and only if *x* = *y*; (2) *d*(*x*,*y*)=*d*(*y*,*x*) (symmetry); (3) d(x,y)≤d(x,z)+d(z,y) (triangle inequality). In a strongly connected directed graph symmetry does not hold in general, while the other two properties remain true. (Strong) connectivity is needed here for otherwise infinite distances get in the way, making the relation with metric spaces improper.

The *(in)-eccentricity* of a node *x* in G is defined as the maximum finite *d*(*y*,*x*) as *y* ranges over all nodes of G. The maximum eccentricity of all nodes in G is called the *diameter* of G, and it is denoted by diam(G).

## 4 Distance-count matrices (DCM)

We start by giving some definitions:

**Definition 4.1 (DCM and CDCM).** *Given a graph* G*, a node x and a natural number k, define*


$$\begin{array}{c} \begin{array}{ccc} N_{G,k}(x)=\{y\in N|d(y,x)=k\} & \; & n_{G,k}(x)=\left|N_{G,k}(x)\right|\\ M_{G,k}(x)=\{y\in N|d(y,x)\leq k\} & \; & m_{G,k}(x)=\left|M_{G,k}(x)\right|. \end{array} \end{array}$$

*The distance-count matrix (DCM) of* G *is the matrix* $N=N_{G}\in R^{n\times n}$ *such that* $n_{i,k}=n_{G,k}(i)$*, i.e., the number of nodes at distance exactly k to i. The cumulative distance-count matrix (CDCM) of* G *is the matrix* $M=M_{G}\in R^{n\times n}$ *such that* $m_{i,k}=m_{G,k}(i)$*, i.e., the number of nodes at distance at most k to i. (It is convenient to assume that vector and matrix elements are indexed starting from zero.)*

**Fig 2:**
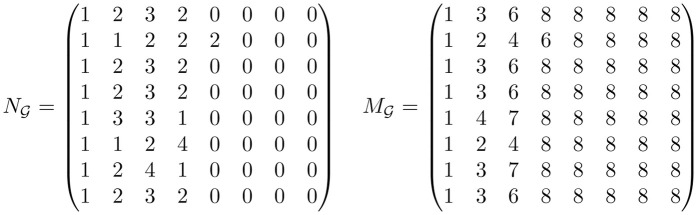
The DCM $N_{G}$ and CDCM $M_{G}$ of the graph G of Fig 1. The highlighted rows correspond to $n_{G,-}(1)$ and $n_{G,-}(3)$.

In Fig 2 we show an example of DCM and CDCM (for the directed graph of Fig 1). Observe that for all *k*,


$$\begin{array}{c} M_{G,k}(x)⧵M_{G,k-1}(x)=N_{G,k}(x) \end{array}$$

where we conveniently assume that $M_{G,-1}(x)=\emptyset$. The definitions describe the set of nodes that are found by moving further and further away from a node. We can think of it as a circular wave that starts from node *x*: as *k* increases we find nodes that are more and more distant from *x*. Eventually, after *k* has reached the eccentricity of *x*, no more nodes are found. We can examine either the set (or count) of nodes at any given distance from the centre (the functions *N* and *n*), or (equivalently) the cumulative set or count of nodes found along the way (the functions *M* and *m*). Also observe that we are computing distances *to x*, not *from x*: this fact is a matter of convention, all our results hold also if we consider the distance from *x* to *y* instead of the distance from *y* to *x*; in the undirected case, the two are the same.

### 4.1 DCMs and linear centralities

A *(graph) centrality* 𝔣 [21] is a function associating with each graph G a map ${𝔣}_{G}:N_{G}\to R$ such that for any two graphs G,H, if φ:G→H is an isomorphism then


$$\begin{array}{c} {𝔣}_{G}(i)={𝔣}_{H}(\varphi (i)) \end{array}$$

for all $i\in N_{G}$.

Centralities that depend essentially only on the rows of the DCM are called *geometric* [7,22]; within this class, particularly relevant are the so-called linear geometric centralities.

Let $𝐚\in R^{N}$: we view **a** as an infinite vector of real numbers. If $A\in R^{m\times n}$ is an m×n matrix, we write A·𝐚 as a shortcut for


$$\begin{array}{c} A\cdot 𝐚[:\;n] \end{array}$$

where 𝐚[:n] stands for the vector of the first *n* entries of **a**.

**Definition 4.2 (Linear (geometric) centrality).** *Given* $𝐚\in R^{N}$ *(the coefficient vector), the centrality* ${𝔏}_{G}^{𝐚}$ *is defined by*


$$\begin{array}{c} {𝔏}_{G}^{𝐚}(i)={\left(N_{G}\cdot 𝐚\right)}_{i}. \end{array}$$

*A centrality is* strictly linear (geometric) *if it is* ${𝔏}_{G}^{𝐚}$ *for some* $𝐚\in R^{𝐍}$*. A centrality is* linear (geometric) *if it is equivalent to (i.e., it produces the same node ranking as) a strictly linear (geometric) centrality.*

One special instance of (strictly) linear centralities are the so-called exponential-decay centralities [23]:

**Definition 4.3 (Exponential-decay centrality).** *Given* δ∈(0,1)*, the* exponential-decay centrality *of a node* $i\in N_{G}$ *is defined by*


$$\begin{array}{c} {𝔈}_{G}^{\delta }(i)={𝔏}_{G}^{𝐚}(i), \end{array}$$

*where* **a** *is obtained for every k > 0 as* $a_{k}=\delta^{k}$*, and a*_*0*_ *= 0*.

Observe that ${𝔈}_{G}^{\delta }(i)$, seen as a function of δ, is a polynomial whose coefficients are those in the *i*-th row of $N_{G}$ (lowest-degree first). We use $P_{G}^{i}$ to denote such a polynomial. More explicitly,


$$\begin{array}{c} P_{G}^{i}(x)=\sum_{t>0}n_{G,t}(i)x^{t}. \end{array}$$

The degree of $P_{G}^{i}$ is at most $|N_{G}|-1.$

### 4.2 Graphical sequences for undirected graphs

A good reference about distances in graphs is [24]: this book includes material on DCMs under different terminology for the undirected case. The *distance degree sequence* (or *dds*) of node *v* of an undirected graph (see [24, Section 9.2]) is the vector


$$\begin{array}{c} dds(v)=(d_{0}(v),d_{1}(v),\ldots ,d_{e(v)}(v)) \end{array}$$

where $d_{i}(v)$ is the number of nodes *w* such that *d*(*v*,*w*) = *i*, and *e*(*v*) is the *eccentricity* of *v*, which is the maximum value of *d*(*v*,*w*) for w∈G. Thus, *dds*(*v*) is row *v* of the DCM, truncated to remove the final zeros.

The *distance degree sequence (or dds)* dds(G) of the graph G is the list of all *dds*’s of its nodes, listed including multiplicity (see [24], Sect 9.2). This is essentially the DCM with zero entries removed.

Also of interest is the *degree sequence* of an undirected graph G (see [24, Section 9.2]), which is the list of degrees of nodes arranged in nonincreasing order. Up to permutation of the nodes, this is column 1 of the DCM of G.

Not all sequences of positive integers can be a degree sequence. A *graphical degree sequence* is a degree sequence that can be realised by an undirected graph. A complete characterization of graphical degree sequences for undirected graphs is given by the Erdős-Gallai theorem:

**Theorem 4.4 ([15]).** *Consider a sequence* $d_{1}\geq d_{2}\geq \ldots \geq d_{p}$ *of p positive integers. This sequence is a graphical degree sequence if and only if its sum is even and for every* n=1,…,p−1


$$\begin{array}{c} \sum_{k=1}^{n}d_{k}\leq n(n-1)+\sum_{k=n+1}^{p}\min(n,d_{k}). \end{array}$$ (1)

The Havel-Hakimi algorithm [16,17] provides a constructive way to check in polynomial time if a sequence is a graphical degree sequence. The algorithm is essentially described by the following [24, Theorem 9.1]:

**Theorem 4.5.** *The sequence* $D=(d_{1},\ldots ,d_{p})$ *with* $p-1\geq d_{1}\geq d_{2}\geq \cdots \geq d_{p}$ *for positive integers* $d_{i}$ *is a graphical degree sequence if and only if*


$$\begin{array}{c} D^{\prime }=(d_{2}-1,d_{3}-1,\ldots d_{d_{1}+1}-1,d_{d_{1}+2},\ldots ,d_{p}), \end{array}$$

*when re-ordered in nonincreasing order, is a graphical degree sequence.*

The Havel-Hakimi algorithm applies Theorem 4.5 inductively: if the process stops at the empty sequence, the original sequence is a graphical degree sequence; if any entry becomes negative before this happens, it is not a graphical degree sequence.

As explained above, column 1 of the DCM of an undirected graph is always a graphical degree sequence. This fact is more general: all columns of a DCM are graphical degree sequences; more precisely, column *k* of the DCM of an undirected graph G is the degree sequence of the graph $G^{k}$ having the same vertices as G and with an edge i↔j iff $d_{G}(i,j)=k$.

### 4.3 Graphical in-degree sequences

*Mutatis mutandis*, we can define the *in-degree sequence* of a directed graph G as the list of in-degrees of nodes arranged in non-increasing order. Up to permutation of the nodes, this is column 1 of the DCM of G. We can ask whether the algorithm described in Theorem 4.5 can be adapted to in-degree sequences. In fact, the result for directed graphs is much simpler:

**Theorem 4.6.** *The sequence* $D=(d_{1},\ldots ,d_{p})$ *with* $p-1\geq d_{1}\geq d_{2}\geq \cdots \geq d_{p}$ *for natural numbers* $d_{i}$ *is always the in-degree sequence of a directed graph.*

*Proof.* Just use N={1,2,…,p} and for every i∈N, add $d_{i}$ edges, whose head is node *i* and whose tails are $d_{i}\leq p-1$ distinct but arbitrarily chosen elements of N⧵{i}. □

A more sophisticated question is whether a given sequence of pairs is the sequence of in- and out-degrees of a graph: the Kleitman–Wang Algorithm [18] extends the Havel-Hakimi Algorithm for this case.

### 4.4 Some basic properties of (C)DCMs

In this brief section we collect some observations about (C)DCMs. Let us start with some trivial ones.

**Proposition 4.7.** *In any graph* G *with n nodes, for any node i of* G*, and for any* 0≤p≤n−1*:*

(a) $M_{0}(i)=N_{0}(i)=\{i\}$.
(b) $m_{0}(i)=n_{0}(i)=1$.
(c) $m_{1}(i)=\nu (i)+1$ *and* $n_{1}(i)=\nu (i)$ *where* ν(i) *is the in-degree (number of predecessors) of i.*
(d) $M_{p}(i)\subseteq M_{p+1}(i)$.
(e) $m_{p}(i)\leq m_{p+1}(i)$.
(f) $n_{p}(i)=m_{p}(i)-m_{p-1}(i)$.
(g) $m_{p}(i)=n_{0}(i)+n_{1}(i)+\cdots +n_{p}(i)$.
(h) *If M is a DCM (resp. CDCM), so is any matrix obtained by permuting the rows of M.*
(i) $m_{n-1}(i)=n$ *if and only if d(j,i) is finite for all nodes j; in particular, this is always true if* G *is strongly connected.*

Observe that the (C)DCM of a graph is uniquely defined only up to row permutation, but by (h) we can use lexicographic order to define a unique (C)DCM of a graph, that we shall call the *canonical* (C)DCM of G.

We can extend the function $M_{p}(-)$ to subsets: for X⊆N, define the *p-neighbourhood* of X to be


$$\begin{array}{c} M_{p}(X)={⋃}_{i\in X}M_{p}(i) \end{array}$$

Clearly


$$\begin{array}{c} M_{p}(M_{q}(X))=M_{p+q}(X). \end{array}$$ (2)

**Proposition 4.8.** *For any graph* G *and node i:*

(a) *If* $m_{p+1}(i)=m_{p}(i)$ *then* $m_{p+r}(i)=m_{p}(i)$ *for* 0≤r≤n−p*.*
(b) *If* G *is strongly connected and* $m_{p+1}(i)=m_{p}(i)$ *then* $m_{p}(i)=n$ *and* $m_{p+r}(i)=n$ *for all* 0≤r≤n−p−1*.*

*Proof.* (a) If $m_{p}(i+1)=m_{p}(i)$ then case (d) of Proposition 4.7 implies that $M_{p+1}(i)=M_{p}(i)$. We use induction on *r*. The result holds for *r* = 1. For the induction step:


$$\begin{array}{c} M_{p+(r+1)}(i)=M_{1}(M_{p+r}(i))=M_{1}(M_{p}(i))=M_{p+1}(i)=M_{p}(i). \end{array}$$

(b) This follows from (a), since $m_{n-1}(i)=n$ for any *i* by connectedness of G.

**Corollary 4.9.** *For any graph* G *and node i the sequence* $\left[m_{p}(i)\right]$ *for* 0≤p≤n−1 *(which is the i-th row of the CDCM) is monotonic strictly increasing until it reaches a certain value k, at which point all subsequent entries equal k. The value k is exactly the number of nodes j such that d(j,i) is finite. If* G *is strongly connected, k = n.*

**Definition 4.10 (Good sequence).** *For a given n, a sequence* $\left(a_{0},a_{1},\ldots ,a_{n-1}\right)$ *of positive integers is good if a*_*0*_ *= 1 and there exists j and* k≤n *such that* $a_{0}<a_{1}<\cdots <a_{j}=k$ *and* $a_{r}=k$ *for all* r≥j*. If further k = n, we say that the sequence is very good.*

Corollary 4.9 states that every row of a CDCM is good. If furthermore the graph is strongly connected, its rows are very good. We observe:

**Theorem 4.11.** *Every good sequence occurs in the CDCM of some graph (in particular, some undirected graph).*

**Fig 3:**
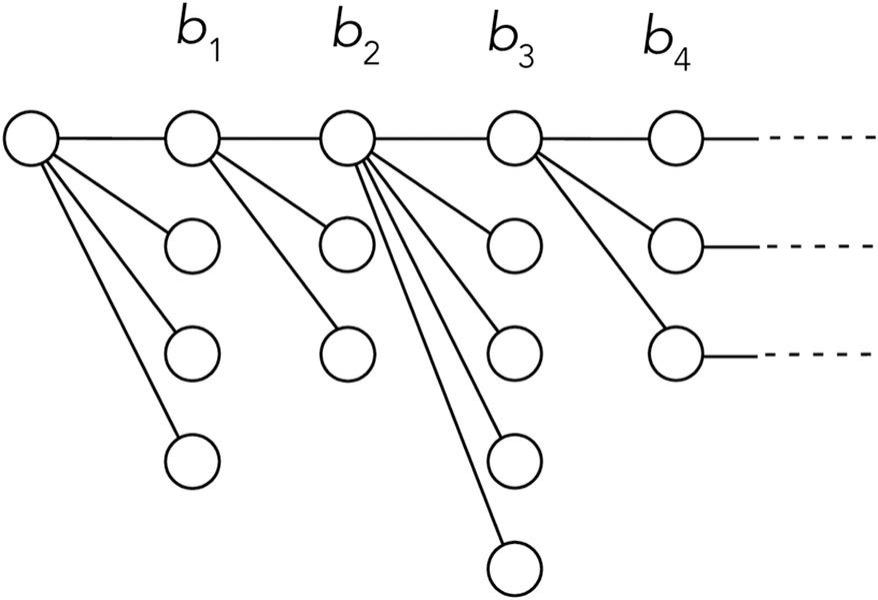
Construction for a graph with a given good row.

*Proof.* Let the good sequence be $\left(a_{0},a_{1},\ldots ,a_{n-1}\right)$, and define $b_{i}=a_{i}-a_{i-1}\geq 0$ for all *i* > 0, and *b*_0_ = 1. Define G to be a tree with root node 0. Node 0 is connected to nodes $1,\ldots ,b_{1}$. One of those nodes connects to nodes $b_{1}+1,\ldots ,b_{1}+b_{2}$, and so on inductively. Then the 0-th row of $N_{G}$ is $\left(b_{0},b_{1},\ldots b_{n-1}\right)$, so the 0-th row of $M_{G}$ is $\left(a_{0},a_{1},\ldots a_{n-1}\right)$. See Fig 3. □

We end this section with some necessary conditions for a matrix to be a CDCM, in terms of inequalities. We already know:

**Theorem 4.12.** *For the CDCM of a strongly connected graph:*

(a) *The column 0 entry in any row is 1.*
(b) *The column 1 entry in row i is* $m_{1}(i)=1+\nu (i)$*. That is, the CDCM (hence also DCM) tells us the in-degree of node i.*
(c) *Each row is very good.*

We now prove two further inequalities.

**Theorem 4.13.** *Let* $M=[m_{j}(i){]}_{i,j}$ *be the CDCM of a graph* G*. Then for any* i∈N*, with* $\nu =\nu (i)=m_{1}(i)-1$*, there exist distinct* $j_{1},\ldots ,j_{\nu }\in N⧵\{i\}$ *such that:*


$$\begin{array}{c} m_{p}(i)\geq \max_{k=1}^{\nu }m_{p-1}(j_{k}) \end{array}$$ (3)

*and*


$$\begin{array}{c} m_{p}(i)\leq \sum_{k=1}^{\nu }m_{p-1}(j_{k}) \end{array}$$ (4)

*for all* p=2,…,n−1.

*Proof.* Let $j_{1},\ldots ,j_{\nu }$ be the predecessors of *i* in G. To prove (3), observe that since $j_{k}\to i$, we have $M_{p-1}(j_{k})\subseteq M_{p}(i)$. Therefore $m_{p}(i)\geq m_{p-1}(j_{k})$. This holds for all *k* such that 1≤k≤ν, and this proves (3). To prove (4), observe that by (2),


$$\begin{array}{c} M_{p}(i)=M_{p-1}(M_{1}(i))={⋃}_{j\to i}M_{p-1}(j). \end{array}$$

Therefore


$$\begin{array}{c} m_{p}(i)\leq \sum_{j\to i}m_{p-1}(j), \end{array}$$

which is (4). □

That is, the vector of entries of row *i* after the first is bounded below, termwise, by the maximum of ν other distinct rows shifted one place to the right. It is bounded above, termwise, by the sum of the same ν distinct rows, again shifted one place to the right. Of course, we do not know which ν rows to choose, but one such set of rows must exist. If no such set exists, the matrix under consideration cannot be a CDCM.

## 5 NP-Completeness of (C)DCM recognizability

In this section, we show that the problem of deciding whether a matrix is a (C)DCM of an undirected graph is (strongly) NP-complete. We use a reduction from a special instance of the *three-partition problem* (TPP) [25], which is a well-known strongly NP-complete problem.

As already discussed, Erdős and Miklós proved in [19] (in our terminology) that deciding whether two sequences of integers are the columns of indices 1 and 2 of the DCM of a simple undirected graph G (the so-called second-order ds problem) is also strongly NP-complete. Their proof uses the *basket filling problem*; more precisely, they prove that the basket filling problem is reducible to the second-order ds problem, while the *three-partition problem* is reducible to the second-order ds problem for bipartite graphs. They also show that the three-partition problem can be reduced in polynomial time to the basket filling problem.

As we already mentioned, their reductions do not immediately apply to the (C)DCM recognizability problem, though. A reduction from the second-order ds problem to the (C)DCM recognizability problem would require that we are able, from a given second-order ds instance, to construct a matrix that is the (C)DCM of some graph. It is interesting to observe that our construction also uses the three-partition problem, but in a simpler way than in Erdős and Miklós’s reduction.

### 5.1 The three-partition problem

Informally, the three-partition problem (shortened hereafter as TPP) consists in deciding if it is possible to partition a list of natural numbers into groups of exactly three integers each, all having the same sum. This problem is very hard: it is strongly NP-complete (i.e., NP-complete even when the input sequence is provided in unary), and it remains so in many restricted cases.

**Definition 5.1 (TPP instance).** *A TPP instance is a sequence of natural numbers* $\langle a_{1},\ldots ,a_{3m}\rangle$ *such that* $a_{1}\geq a_{2}\geq \ldots \geq a_{3m}$ *and* $t/4<a_{i}<t/2$ *for all* i=1,…,3m*, where* $t=(\sum_{i=1}^{3m}a_{i})/m$*.*

Formally, the three-partition problem is

**Problem 5.2 [Three-partition problem (TPP)].** *Given a TPP instance* $\langle a_{1},\ldots ,a_{3m}\rangle$*, decide whether there exist subsets* $D_{1},\ldots ,D_{m}\subseteq \{1,\ldots ,3m\}$ *such that:*

– *the* $D_{j}$*’s are pairwise disjoint;*
– $|D_{j}|=3$ *for all* j=1,…,m;
– $\sum_{i\in D_{j}}a_{i}=t$ *for all* j=1,…,m,

*where* $t=(\sum_{i=1}^{3m}a_{i})/m$*. If this partition exists, the instance is* positive*, otherwise it is* negative*.*

We consider a very mild variant, where we assume that all integers are distinct and separated from one another by a certain threshold. This variant is still strongly NP-complete, as shown by the following result.

**Theorem 5.3.** *Problem 5.2 (TPP) is strongly NP-complete (i.e., it is NP-complete even if the input sequence is assumed to be encoded in unary); it remains so even under the following further assumptions:*

– *all* $a_{i}$*’s are distinct,*
– t≥4,
– *for a fixed positive K,* $a_{3m}\geq K$ *and* $a_{i}-a_{i+1}\geq K$ *for all* i=1,…,3m−1.

*Proof.* The problem is strongly NP-complete as shown in [25]. It remains so even if the input integers are all distinct, as proved in [26]; the fact that the latter result holds also in the presence of the bounds $t/4<a_{i}<t/2$ is easy (by adding 2*t* to each entry in the sequence).

If $t=(\sum_{i=1}^{3m}a_{i})/m\leq 3$, then all $a_{i}$’s must be zero or one (because $a_{i}<t/2\leq 3/2$), so the instance is negative because it contains only one or two elements (recall that the elements in the sequence are all distinct).

Hence, the problem remains strongly NP-complete for distinct integers with t≥4. Note that in this case $a_{i}>t/4\geq 1$, so all $a_{i}$ are strictly positive. Multiplying all the entries by *K* we can assume that $a_{3m}\geq K$ and $a_{i}-a_{i+1}\geq K$ for all i=1,…,3m−1 (multiplying all elements by a constant does not change the problem, because the sequence obtained after the multiplication is a positive instance of the problem if and only if the original sequence was itself a positive instance). □

As a consequence, we can assume that the input sequence of a TPP instance is a TPP instance in the sense of the following definition, which includes all the assumptions of Theorem 5.3 for *K* = 3.

**Definition 5.4 (Special TPP instance).** *A special TPP instance is a TPP instance* $\langle a_{1},\ldots ,a_{3m}\rangle$ *with the following additional properties:* $a_{3m}\geq 3$*,* $a_{i}-a_{i+1}\geq 3$ *(for all* i=1,…,3m−1*) and* $t=(\sum_{i=1}^{3m}a_{i})/m\geq 4$*.*

### 5.2 NP-Completeness of (C)DCM

Given a special TPP instance $𝐚=\langle a_{1},\ldots ,a_{3m}\rangle$, let


$$\begin{array}{c} s=\sum_{i=1}^{3m}a_{i}\;t=s/m\;n=4m+s=(t+4)m. \end{array}$$

We define a matrix $M(𝐚)\in R^{n\times n}$ as follows:

– the first 3*m* rows of M(𝐚) have the form $\left[1,a_{i},1,t-a_{i},2,0,0,\ldots \right]$ for i=1,…,3m,
– the following *s* rows of M(𝐚) have the form [1,2,t−1,2,0,0,0,…],
– the last *m* rows of M(𝐚) have the form [1,t,3,0,0,0,…].

**Fig 4:**
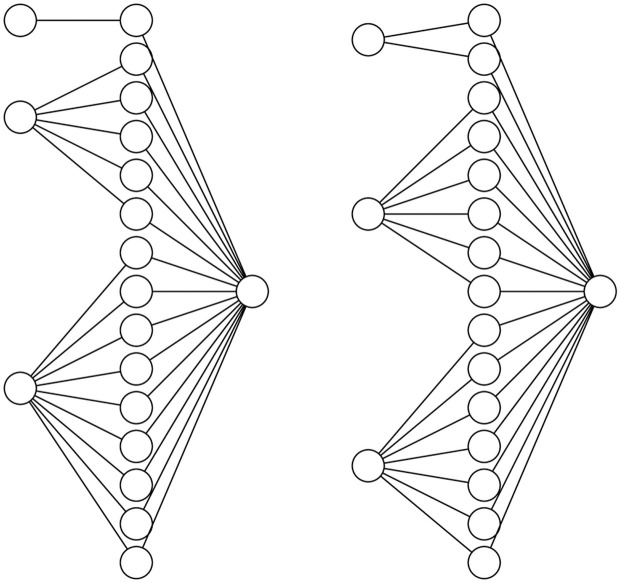
The graph G(𝐚) of the construction of Theorem 5.5, for the sequence 𝐚=⟨9,7,6,5,2,1⟩; recall that this is a positive instance of TPP (without the further assumptions of Theorem 5.3), because 1 + 5 + 9 = 2 + 6 + 7 = 15.

Intuitively, if **a** is a positive instance of TPP, it is possible to show that M(𝐚) is the DCM of an undirected graph G(𝐚). This fact will be part of the proof of Theorem 5.5, but we want to provide immediately a visual clue to this fact for 𝐚=⟨9,7,6,5,2,1⟩. To keep the graph small, we use a sequence that fails to satisfy the more restrictive conditions of Theorem 5.3. The graph (see Fig 4) contains *m* (in this case, 2) connected components, one for each $D_{j}$. All components contain three vertices (representing the three integers of the input sequence belonging to $D_{j}$) connected to *t* vertices (this is possible because the sum of the three integers is always *t*), which are further connected to a single vertex (of degree *t*).

**Theorem 5.5.** *Given a special TPP instance* $𝐚=\langle a_{1},\ldots ,a_{3m}\rangle$*, the following two statements are equivalent:*

1. **a** *is a positive instance of Problem 5.2;*
2. M(𝐚) *is the DCM of an undirected graph.*

*Proof.* (1) ⟹ (2). Let $D_{1},\ldots ,D_{m}$ be a partition of {1,…,3m} showing that the sequence is a positive instance. Build an undirected graph G(𝐚) with *n* vertices as follows:

– there is one vertex for every integer in the sequence **a**; we will call these vertices $x_{1},\ldots ,x_{3m}$;
– there are *t* vertices for every class $D_{j}$; we will call them $y_{1}^{j},\ldots ,y_{t}^{j}$ for each j=1,…,m;
– there is one vertex $z_{j}$ for every class $D_{j}$, j=1,…,m;
– for every j=1,…,m, suppose $D_{j}=\{i_{1},i_{2},i_{3}\}$: we add edges
  - $x_{i_{1}}↔y_{u}^{j}$ for every $u=1,\ldots ,a_{i_{1}}$;
  - $x_{i_{2}}↔y_{u}^{j}$ for every $u=a_{i_{1}}+1,\ldots ,a_{i_{1}}+a_{i_{2}}$;
  - $x_{i_{3}}↔y_{u}^{j}$ for every $u=a_{i_{1}}+a_{i_{2}}+1,\ldots ,a_{i_{1}}+a_{i_{2}}+a_{i_{3}}=t$;
– for every j=1,…,m and u=1,…,t, we add an edge $y_{u}^{j}↔z_{j}$.

Each vertex $x_{i}$ has degree $a_{i}$, and each vertex $y_{u}^{j}$ has degree 2 (because it is connected to $x_{i}$ for exactly one $i\in D_{j}$, and to $z_{j}$). Each $z_{j}$ has degree *t*. All vertices related to *j* (i.e., $x_{i}$ for $i\in D_{j}$, $y_{u}^{j}$ for u=1,…,t, and $z_{j}$) form one connected component with *t* + 4 vertices. There are *m* such components.

If $i\in D_{j}$, $x_{i}$ has only vertex $z_{j}$ at distance two, and $t-a_{i}$ vertices at distance three (all the vertices of the form $y_{u}^{j}$ except those that are neighbors of $x_{i}$). Finally, $x_{i}$ has two vertices at distance four (exactly the other two vertices of the form $x_{p}$ for $p\in D_{j}$, and p≠i: there are two of them, because $|D_{j}|=3$). Hence the row related to $x_{i}$ is of the form $\left[1,a_{i},1,t-a_{i},2,0,0,\ldots \right]$.

The vertices at distance two from $y_{u}^{j}$ are all the other $y_{w}^{j}$ (there are t−1 of them). The vertices at distance three are 2 (the vertices of the form $x_{p}$ for $p\in D_{j}$, except the only one that is a direct neighbor of $y_{u}^{j}$). Hence, the row related to $y_{u}^{j}$ is of the form [1,2,t−1,2,0,0,0,…].

Finally, the vertices at distance two from $z_{j}$ are all the vertices of the form $x_{i}$ for $i\in D_{j}$ (there are 3 of them). Thus, the row related to $z_{j}$ is of the form [1,t,3,0,0,0,…].

It is immediate to check that the DCM of G(𝐚) is M(𝐚), meaning that M(𝐚) is a DCM.

(2) ⟹ (1). Suppose that M(𝐚) is the DCM of some undirected graph G. The graph has *n* vertices, divided into three groups:

– Group I: the first 3*m* vertices all have degree larger than 2 (because **a** is a TPP instance such that all of its elements are ≥3); group I corresponds to rows of the form $\left[1,a_{i},1,t-a_{i},2,0,\ldots \right]$;
– Group II: the next *s* vertices all have degree 2; group II corresponds to rows of the form [1,2,t−1,2,0,…];
– Group III: the last *m* vertices all have degree *t* (which is larger than *a*_1_, hence of all $a_{i}$’s, because $a_{i}<t/2$); group III corresponds to rows of the form [1,t,3,0,…];

Observe that every row of M(𝐚) has sum equal to *t* + 4. Hence the graph has *m* connected components, wi*t*h *t* + 4 vertices each. It is also worth *t*aking note of the form of the rows of the CDCM of G:

– Group I: $\left[1,a_{i}+1,a_{i}+2,t+2,t+4,t+4,\ldots \right]$,
– Group II: [1,3,t+2,t+4,t+4,t+4,…],
– Group III: [1,t+1,t+4,t+4,t+4,t+4,…].

Consider any component *X* of G, and suppose that this component contains *A* vertices of group I, *B* vertices of group II and *C* vertices of group III. The degree sequence of this component is (after reordering):


$$\begin{array}{c} \left[\underset{C}{\underbrace{t,t,\ldots ,t}},\underset{A}{\underbrace{a_{i_{1}},\ldots ,a_{i_{A}}}},\underset{B}{\underbrace{2,\ldots ,2}}\right] \end{array}$$

for some choice of $i_{1}>i_{2}>\ldots >i_{A}$. Note that *A* + *B* + *C* = *t* + 4 (the size of each component).

Start by considering a vertex i∈X of group II: it is connected to two vertices *j*_1_ and *j*_2_, which may each belong, in principle, to one of the three groups. We shall apply Theorem 4.13 to the row of vertex *i* in the CDCM of G: Table 1 describes the possible intervals of integers for the third value in the row of *i* depending on how we choose *j*_1_ and *j*_2_.

**Table 1:** The third value in the row of node *i* of group II depending on the group its two in-neighbors *j*_1_ and *j*_2_ belong to. Here, we are assuming without loss of generality that $a_{h}>a_{k}$.

*j*_2_ (degree) *j*_1_ (degree)	Group I ($a_{k}$)	Group II (2)	Group III (*t*)
Group I ($a_{h}$)	$a_{h}+1\ldots a_{h}+a_{k}+2$	$a_{h}+1\ldots a_{h}+4$	$t+1\ldots a_{h}+t+2$
Group II (2)		3…6	t+1…t+4
Group III (*t*)			t+1…2t+2

The third value in the row of the CDCM for group II is *t* + 2, and the only intervals containing *t* + 2 are those in the last column (since $a_{3m}\geq 3$, *t* is certainly larger than 6). This means tha*t* every vertex of group II is connected to at least one vertex of group III. As a consequence, *B* > 0 implies *C* > 0.

On the other hand, applying again Theorem 4.13, no vertex i∈X of group I can be connected to a node of group III, because $t+1>a_{i}+2$. Also, no two vertices of group I can be connected to each other: suppose, by contradiction that a vertex of degree $a_{h}$ is connected to a vertex of degree $a_{k}$ with $a_{h}>a_{k}$. Then we should have (by Theorem 4.13) $a_{h}+1\leq a_{k}+2$, that is $a_{h}-a_{k}\leq 1$, which is impossible because $a_{h}-a_{k}\geq 3$. We conclude that vertices of group I are connected only to vertices of group II. As a consequence, *A* > 0 implies *B* > 0.

For the reasons above, every component of G must contain at least one vertex of group III. Since there are *m* components and exactly *m* vertices of group III, we conclude that each component contains *exactly one* vertex of group III, that is, *C* = 1.

The *A* vertices of group I in component *X* are all connected to vertices of group II (this is, as we discussed above, the only possibility for them), and those vertices of group II must be distinct (they have degree 2, and each of them is connected to at least one vertex of group III); so there must be at least $a_{i_{1}}+\ldots +a_{i_{A}}$ vertices of group II in the component, say $a_{i_{1}}+\ldots +a_{i_{A}}+e_{X}$ for some $e_{X}\geq 0$. Assume for the moment that $e_{X}=0$.

The only vertex of group III is itself connected to *t* vertices of group II, so we conclude that $a_{i_{1}}+\ldots +a_{i_{A}}=t$ and *B* = *t*. Since *A* + *B* + *C* = *t* + 4, we have *A* + *t* + 1 = *t* + 4, hence *A* = 3.

These conditions, together with the fact that this property holds for all components of G, allow us to conclude that **a** is a positive instance of TPP.

Finally, note that $e_{X}$ *must* be zero, because if even one component contained some extra vertex of group II, then altogether there would be more than *s* vertices of group II in the graph, which is impossible. □

**Corollary 5.6.** *Recognizing whether a given matrix is the (C)DCM of an undirected graph is strongly NP-complete.*

*Proof.* Theorem 5.5 reduces TPP to recognizing DCMs, hence the result follows by Theorem 5.3. The fact that TPP is strongly NP-complete is needed for the construction to be admissible (the size of the matrix is pseudopolynomial in the input sequence **a**). □

Observe that our construction does not directly tell us if the problem of recognizing whether a matrix is the (C)DCM of an *arbitrary* directed graph is also NP-complete. Incidentally, it is easy to see that 𝐚=⟨41,37,34,31,28,25⟩ is a *negative* instance of TPP satisfying the conditions in the statement of Theorem 5.3: still, we were able to find a *directed* graph whose DCM is M(𝐚). In other words, the construction we exhibited is not enough to conclude that recognizing general (C)DCMs is itself an NP-complete problem, although we believe so.

## 6 Graph operations and (C)DCMs

In this section, we want to show that, while the recognition problem of (C)DCMs is extremely hard, distance-count matrices exhibit a rich algebraic structure that matches natural operations between the underlying graphs. This fact can be used as the starting point for obtaining positive and negative results about DCMs, by exploiting this modular correspondence. We start with a precise definition of what we want to describe:

**Definition 6.1 (DCM-stable graph operation).** *A graph operation* ⊗ *is DCM-stable if there is a matrix operation* ⊛ *such that, for any two graphs* G *and* H *we have*


$$\begin{array}{c} N_{G⊗H}=N_{G}⊛N_{H}. \end{array}$$

*It is DCM-stable on undirected graphs if the above equality occurs only for undirected graphs* G, H.

Note that here and in the following, the identity of (C)DCM matrices is intended up to row permutations. Checking whether *A* = *B* can be accomplished by sorting the rows of each matrix in lexicographic order, and then checking if the resulting matrices are identical.

We will show that many common graph operations are DCM-stable. In particular, we will consider disjoint union, subgraphs, and different types of graph products. In the rest of the paper, all results apply to general directed graphs, unless otherwise specified.

### 6.1 Disjoint union

The disjoint union of graphs is possibly the simplest binary graph operation: it consists of taking the actual union of nodes and arcs, without any extra arcs added between the two graphs. Since the two node sets may partially overlap, it is formally necessary to label each node so that we know where it comes from:

**Definition 6.2 (Disjoint union).** *Given graphs* G *and* H*, their disjoint union* G⊕H *has node set* $N_{G}\times \{0\}\cup N_{H}\times \{1\}$ *and contains an arc ((x,0),(y,0)) for every arc* $(x,y)\in E_{G}$*, and an arc ((x,1),(y,1)) for every arc* $(x,y)\in E_{H}$*.*

It is nonetheless convenient (and in fact equivalent, up to node renaming) to assume that $N_{G}$ and $N_{H}$ are disjoint sets, and to simply let


$$\begin{array}{c} N_{G⊕H}=N_{G}\cup N_{H} \end{array}$$

$$\begin{array}{c} E_{G⊕H}=E_{G}\cup E_{H}. \end{array}$$

**Fig 5:**
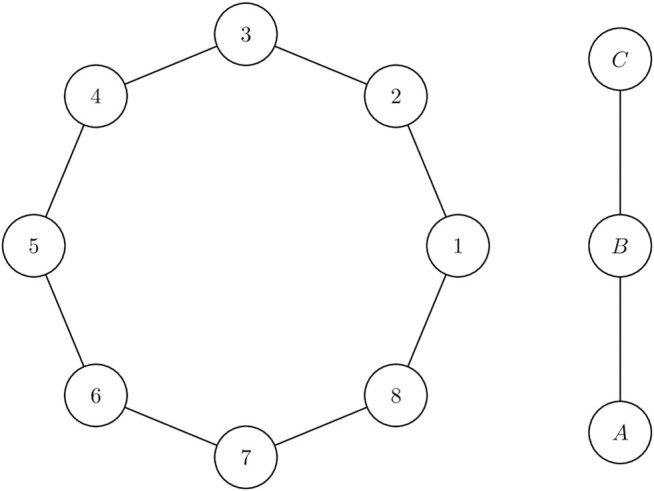
The graph G⊕H, where G is an undirected 8-cycle and H is an undirected 3-path.

Now paths (hence, shortest paths) in G⊕H are simply paths in the two original graphs, which implies that (Fig 5):

**Proposition 6.3** *Let* G *and* H *be two graphs, and* G⊕H *denote their disjoint union. Then, for all* $x,y\in N_{G⊕H}$ *we have:*


$$\begin{array}{c} d_{G⊕H}(x,y)=\left\{ \begin{array}{cc} d_{G}(x,y) & \text{if }x,y\in N_{G}\\ d_{H}(x,y) & \text{if }x,y\in N_{H}\\ \infty & \text{otherwise}. \end{array}\right. \end{array}$$

Hence:

**Theorem 6.4** *Let* G *and* H *be two graphs, and* G⊕H *denote their disjoint union. Then for every* $x\in N_{G⊕H}$ *and natural number k,*


$$\begin{array}{c} n_{G⊕H,k}(x)=\left\{ \begin{array}{cc} n_{G,k}(x) & \text{if }x\in N_{G}\\ n_{H,k}(x) & \text{if }x\in N_{H}. \end{array}\right. \end{array}$$

**Corollary 6.5** *Disjoint union* ⊕ *of graphs is DCM-stable.*

### 6.2 Standard graph products

Graph products [13], in general, are binary graph operations between a graph G and another graph H that yield a graph whose node set is the cartesian product $N_{G}\times N_{H}$. The arcs in the product can be defined in different ways, yielding different forms of graph product. It is often useful to think of the arcs in G and H as possible “moves in a game”, and the product as a way to compose those moves. In particular, [13, Chapter 5] considers four so-called *standard products*: strong, cartesian, lexicographic, and Kronecker product. Note that some results about distance degree sequences in these products are already known in the literature [27–30]; here we will extend those results to the full distance-count matrix.

#### 6.2.1 Strong product.

**Fig 6:**
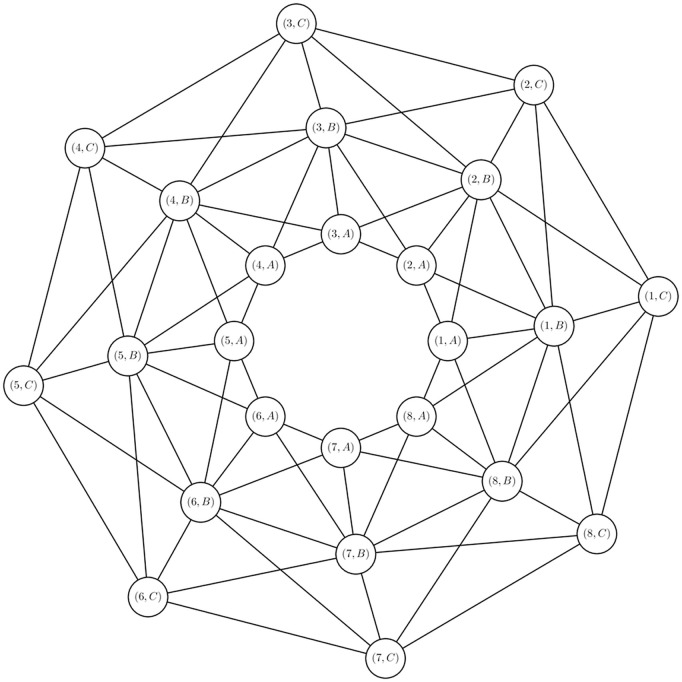
The strong product G⊠H, where G is an undirected 8-cycle and H is an undirected 3-path (see Fig 5).

Let us start with the strong product (see Fig 6). Here, moves in the product correspond to moves in at least one graph (possibly both). The following is a formal definition:

**Definition 6.6 (Strong product).** *Given graphs* G *and* H*, their strong product* G⊠H *has node set* $N_{G}\times N_{H}$ *and contains an arc* $\left((x,y),(x^{\prime },y^{\prime })\right)$ *if and only if one of the following is true:*

- $x=x^{\prime }$ and $(y,y^{\prime })\in E_{H}$;
- $y=y^{\prime }$ and $(x,x^{\prime })\in E_{G}$;
- $(x,x^{\prime })\in E_{G}$ and $(y,y^{\prime })\in E_{H}$.

A path in G⊠H is a sequence of moves in either graph, or in both of them. More precisely, any path $(x_{1},y_{1}),\ldots ,(x_{k},y_{k})\in E_{G⊠H}$ can be projected onto G (H, respectively) by taking $x_{1},\ldots ,x_{k}$ ($y_{1},\ldots ,y_{k}$, resp.) and removing repeated consecutive occurrences of the same node. Hence, every path of length *k* from (*x*,*y*) to $\left(x^{\prime },y^{\prime }\right)$ in G⊠H yields a path of length at most *k* from *x* to $x^{\prime }$ in G and a path of length at most *k* from *y* to $y^{\prime }$ in H. Hence [13],


$$\begin{array}{c} d_{G⊠H}((x,y),(x^{\prime },y^{\prime }))\geq \max\left(d_{G}(x,x^{\prime }),d_{H}(y,y^{\prime })\right). \end{array}$$

On the other hand, it is easy to see that this is an equality, by taking a path that follows the shortest path from *x* to $x^{\prime }$ in G and the shortest path from *y* to $y^{\prime }$ in H in parallel until this is possible, and then remaining still in either graph and continuing in the other one for the remaining steps. As a consequence:

**Proposition 6.7 (Proposition 5.4 of [13]).** *Let* G *and* H *be two graphs, and* G⊠H *denote their strong product. Then for every* $(x,y),(x^{\prime },y^{\prime })\in N_{G⊠H}$ *we have*


$$\begin{array}{c} d_{G⊠H}((x,y),(x^{\prime },y^{\prime }))=\max\left(d_{G}(x,x^{\prime }),d_{H}(y,y^{\prime })\right). \end{array}$$

Note that this proposition (like other similar ones that we will be using from [13]) holds also in the directed case. A consequence of Proposition 6.7 is the following:

**Theorem 6.8.** *Let* G *and* H *be two graphs, and let* G⊠H *denote their strong product. Then for every* $(x,y)\in N_{G⊠H}$ *and natural number k,*


$$\begin{array}{c} n_{G⊠H,k}((x,y))=m_{G,k}(x)\cdot n_{H,k}(y)+n_{G,k}(x)\cdot m_{H,k}(y)-n_{G,k}(x)\cdot n_{H,k}(y). \end{array}$$

*Proof.* The set *A* of nodes $\left(x^{\prime },y^{\prime }\right)$ such that $d_{G⊠H}((x^{\prime },y^{\prime }),(x,y))=k$ consists of those for which


$$\begin{array}{c} \max\left(d_{G}(x^{\prime },x),d_{H}(y^{\prime },y)\right)=k. \end{array}$$

Consider all the pairs of natural numbers $h_{1},h_{2}$ such that $\max(h_{1},h_{2})=k$: we can characterize *A* as the union of all pairs $\left(x^{\prime },y^{\prime }\right)$ such that $d_{G}(x^{\prime },x)=h_{1}$ and $d_{H}(y^{\prime },y)=h_{2}$. In other words, for $\max(h_{1},h_{2})=k$ we must have either *h*_1_ = *k* or *h*_2_ = *k* (or both). So we can count the cardinality of *A* as follows:


$$\begin{array}{c} n_{G,k}(x)\sum_{h\leq k}n_{H,h}(y)+n_{H,k}(y)\sum_{h\leq k}n_{G,h}(x)-n_{G,k}(x)\cdot n_{H,k}(y), \end{array}$$

where the last summand is needed to remove the pairs that are both at distance *k*. □

**Corollary 6.9.** *Strong product* ⊠ *of graphs is DCM-stable.*

#### 6.2.2 Cartesian product.

**Fig 7:**
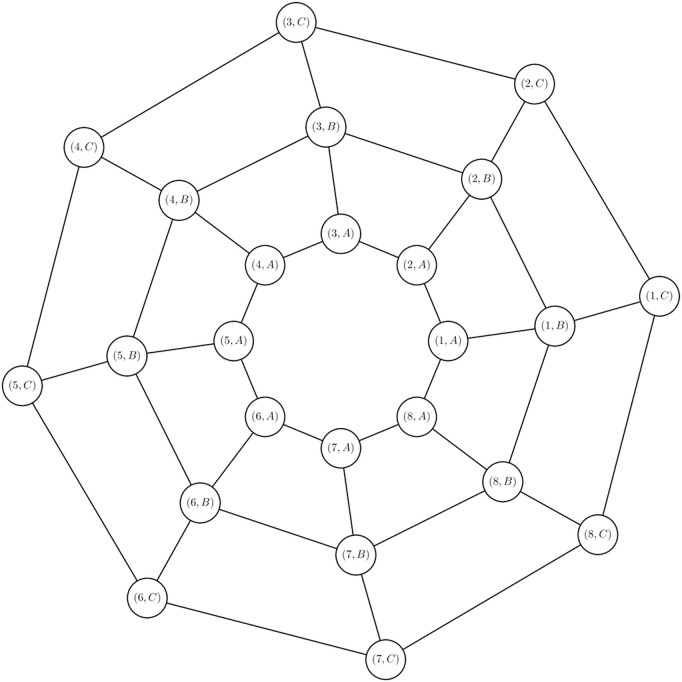
The cartesian product G◻H, where G is an undirected 8-cycle and H is an undirected 3-path (see Fig 5).

The cartesian product (see Fig 7) is a different form of product where moves in the product correspond to moves in one of the graphs, but not both. The following is a formal definition:

**Definition 6.10 (Cartesian product).** *Given graphs* G *and* H*, their cartesian product* G◻H *has node set* $N_{G}\times N_{H}$ *and contains an arc* $\left((x,y),(x^{\prime },y^{\prime })\right)$ *if and only if one of the following is true:*

- $x=x^{\prime }$ and $(y,y^{\prime })\in E_{H}$;
- $y=y^{\prime }$ and $(x,x^{\prime })\in E_{G}$.

Following the same line of reasoning as for the strong product, we have:

**Proposition 6.11 (Proposition 5.1 of [13]).** *Let* G *and* H *be two graphs, and* G◻H *denote their cartesian product. Then for every* $(x,y),(x^{\prime },y^{\prime })\in N_{G◻H}$ *we have*


$$\begin{array}{c} d_{G◻H}((x,y),(x^{\prime },y^{\prime }))=d_{G}(x,x^{\prime })+d_{H}(y,y^{\prime }). \end{array}$$

Interestingly, the converse of this fact is also true

**Proposition 6.12** *Let* G *and* H *be two graphs, and* K *be a graph with* $N_{K}=N_{G}\times N_{H}$*. Suppose that for every* $(x,y),(x^{\prime },y^{\prime })\in N_{K}$ *we have*


$$\begin{array}{c} d_{K}((x,y),(x^{\prime },y^{\prime }))=d_{G}(x,x^{\prime })+d_{H}(y,y^{\prime }). \end{array}$$

*Then* K=G◻H.

*Proof.* We have that $\left(x,y)\to_{K}(x^{\prime },y^{\prime }\right)$ if and only if $d_{K}((x,y),(x^{\prime },y^{\prime }))=1$, which happens if and only if $d_{G}(x,x^{\prime })+d_{H}(y,y^{\prime })=1$. The latter is equivalent to $x\to_{G}x^{\prime }$ and $y=y^{\prime }$, or $x=x^{\prime }$ and $y\to_{H}y^{\prime }$, so K=G◻H.

An immediate consequence of Proposition 6.11 is the following

**Theorem 6.13.** *Let* G *and* H *be two graphs, and* G◻H *denote their cartesian product. Then for every* $(x,y)\in N_{G◻H}$ *and natural number k,*


$$\begin{array}{c} n_{G◻H,k}((x,y))=\sum_{h\leq k}n_{G,h}(x)\cdot n_{H,k-h}(y). \end{array}$$

Recalling the definition of exponential-decay centrality, we have that


$$\begin{array}{c} P_{G◻H}^{\left(x,y\right)}=P_{G}^{x}\cdot P_{H}^{y}, \end{array}$$

hence


$$\begin{array}{c} {𝔈}_{G◻H}^{\delta }((x,y))={𝔈}_{G}^{\delta }(x)\cdot {𝔈}_{H}^{\delta }(y) \end{array}$$

for every δ∈(0,1). More is true:

**Theorem 6.14.** *Let* G *and* H *be two graphs, and* K *be a graph with node set* $N_{G}\times N_{H}$*. Assume moreover that for any* $(x,y)\in N_{K}$ *and for at least* $\left|N_{K}\right|$ *distinct values* δ∈(0,1) *we have*


$$\begin{array}{c} {𝔈}_{K}^{\delta }((x,y))={𝔈}_{G}^{\delta }(x)\cdot {𝔈}_{H}^{\delta }(y). \end{array}$$ (5)

*Then (5) holds for any* δ∈(0,1)*; moreover, for every* $(x,y)\in N_{K}$ *and natural number k,*


$$\begin{array}{c} n_{K}((x,y))=\sum_{h\leq k}n_{G,h}(x)\cdot n_{H,k-h}(y). \end{array}$$

*Proof.* Remember that ${𝔈}_{G}^{\delta }(x)=P_{G}^{x}(\delta )$ and similarly for H and K, so we have


$$\begin{array}{c} P_{K}^{\left(x,y\right)}(\delta )=P_{G}^{x}(\delta )\cdot P_{H}^{y}(\delta ) \end{array}$$

for at least $\left|N_{K}\right|$ values δ∈(0,1). Since on both sides of the equation we have polynomials of degree at most $|N_{K}|-1$, we conclude that those polynomials in fact coincide; that is,


$$\begin{array}{c} P_{K}=P_{G}\cdot P_{H}, \end{array}$$

i.e., for all *t*

$n_{K,t}((x,y))=\sum_{s\leq t}n_{G,s}(x)\cdot n_{H,t-s}(y).$ □

**Corollary 6.15.** *Cartesian product* ◻ *of graphs is DCM-stable.*

#### 6.2.3 Lexicographic product.

**Fig 8:**
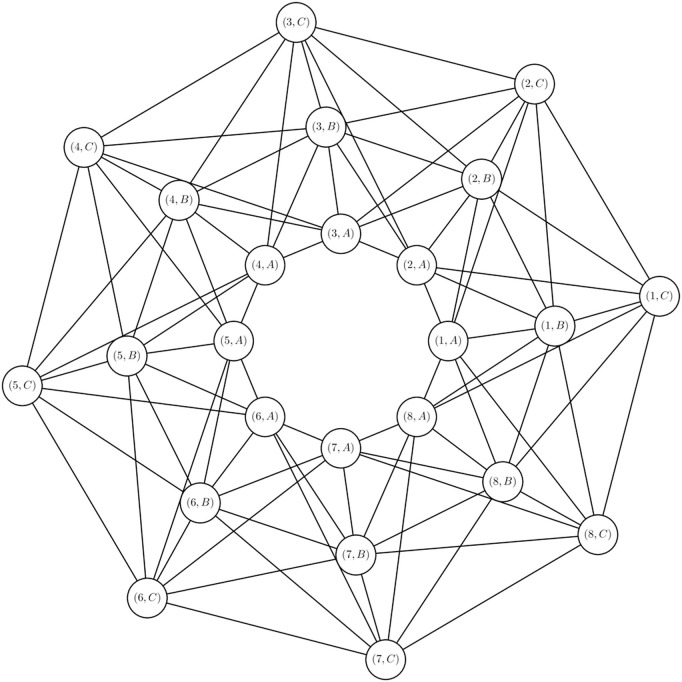
The lexicographic product G∘H, where G is an undirected 8-cycle and H is an undirected 3-path (see Fig 5).

The third common type of graph product [13] is the following (see Fig 8):

**Definition 6.16 (Lexicographic product).** *Given graphs* G *and* H*, their lexicographic product* G∘H *has node set* $N_{G}\times N_{H}$ *and contains an arc* $\left((x,y),(x^{\prime },y^{\prime })\right)$ *if and only if one of the following is true:*

- $(x,x^{\prime })\in E_{G}$;
- $x=x^{\prime }$ and $(y,y^{\prime })\in E_{H}$.

Distances in the lexicographic product can be characterized as follows for the undirected case:

**Proposition 6.17 (Proposition 5.12 of [13]).** *Let* G *and* H *be two undirected graphs, and* $(x,y),(x^{\prime },y^{\prime })\in N_{G\circ H}$*. Then:*


$$\begin{array}{c} d_{G\circ H}((x,y),(x^{\prime },y^{\prime }))=\left\{ \begin{array}{cc} d_{G}(x,x^{\prime }) & \text{if }x\neq x^{\prime }\\ \min\left(d_{H}(y,y^{\prime }),2\right) & \text{if }x=x^{\prime }\text{ and }\deg_{G}(x)>0\\ d_{H}(y,y^{\prime }) & \text{otherwise.} \end{array}\right. \end{array}$$

The directed version of the same property is trickier: the 2 appearing in the lemma would become the length of the shortest cycle of G involving *x*, information that cannot be deduced from the DCM of G itself.

**Theorem 6.18** *Let* G *and* H *be two undirected graphs, and* G∘H *denote their lexicographic product. Then for every* $(x,y)\in N_{G\circ H}$ *and natural number k,*

• *if* $n_{G,1}(x)>0$ *then*

$$\begin{array}{c} n_{G\circ H,k}((x,y))=\left\{ \begin{array}{cc} 1 & \text{if }k=0\\ n_{G,1}(x)|N_{H}|+n_{H,1}(y) & \text{if }k=1\\ n_{G,2}(x)|N_{H}|+|N_{H}|-n_{H,1}(y)-1 & \text{if }k=2\\ n_{G,k}(x)|N_{H}| & \text{if }k>2; \end{array}\right. \end{array}$$

• *else*

$$\begin{array}{c} n_{G\circ H,k}((x,y))=\left\{ \begin{array}{cc} 1 & \text{if }k=0\\ n_{H,k}(y) & \text{otherwise}. \end{array}\right. \end{array}$$

*Proof. Case 1.* Let us consider the case $n_{G,1}(x)=\deg(x)>0$ first. By Proposition 6.17, in this case we have


$$\begin{array}{c} d_{G\circ H}((x,y),(x^{\prime },y^{\prime }))=\left\{ \begin{array}{cc} d_{G}(x,x^{\prime }) & \text{if }x\neq x^{\prime }\\ \min\left(d_{H}(y,y^{\prime }),2\right) & \text{if }x=x^{\prime } \end{array}\right. \end{array}$$

that is, more explicitly,


$$\begin{array}{c} d_{G\circ H}((x,y),(x^{\prime },y^{\prime }))=\left\{ \begin{array}{cc} d_{G}(x,x^{\prime }) & \text{if }x\neq x^{\prime }\\ 0 & \text{if }x=x^{\prime }\text{ and }y=y^{\prime }\\ 1 & \text{if }x=x^{\prime }\text{ and }d_{H}(y,y^{\prime })=1\\ 2 & \text{if }x=x^{\prime }\text{ and }d_{H}(y,y^{\prime })>1. \end{array}\right. \end{array}$$

Now, for any given (*x*,*y*) and natural number *k*, the pairs $\left(x^{\prime },y^{\prime }\right)$ that are at distance *k* from (*x*,*y*) are:

- just (*x*,*y*), if *k* = 0;
- if *k* = 1, we have all the pairs $\left(x^{\prime },y^{\prime }\right)$ with $d_{G}(x,x^{\prime })=1$, for every possible $y^{\prime }\in N_{H}$, plus all the pairs $\left(x,y^{\prime }\right)$ with $d_{H}(y,y^{\prime })=1$;
- if *k* = 2, we have all the pairs $\left(x^{\prime },y^{\prime }\right)$ with $d_{G}(x,x^{\prime })=2$, for every possible $y^{\prime }\in N_{H}$, plus all the pairs $\left(x,y^{\prime }\right)$ with $d_{H}(y,y^{\prime })>1$ (all possible $y^{\prime }\in N_{H}$, except when $y^{\prime }=y$ or $d_{H}(y,y^{\prime })=1$);
- if *k* > 2, we have all the pairs $\left(x^{\prime },y^{\prime }\right)$ with $d_{G}(x,x^{\prime })=k$, for every possible $y^{\prime }\in N_{H}$.

*Case 2*. In the case $n_{G,1}(x)=\deg(x)=0$, then $n_{G,k}(x)=0$ for all *k* > 0. Hence


$$\begin{array}{c} d_{G\circ H}((x,y),(x^{\prime },y^{\prime }))=\left\{ \begin{array}{cc} d_{G}(x,x^{\prime }) & \text{if }x\neq x^{\prime }\\ d_{H}(y,y^{\prime }) & \text{otherwise.} \end{array}\right. \end{array}$$

Now, for any given (*x*,*y*) and natural number *k*, the pairs $\left(x^{\prime },y^{\prime }\right)$ that are at distance *k* > 0 from (*x*,*y*) are those with $d_{G}(x,x^{\prime })=k$ (there are none), plus all those of the form $\left(x,y^{\prime }\right)$ with $d_{H}(y,y^{\prime })=k$.□

**Corollary 6.19.** *Lexicographic product* ∘ *of graphs is DCM-stable for undirected graphs.*

This result is anyway not true for *directed* graphs. To prove that the lexicographic product is *not* DCM-stable, we must provide four digraphs G, $G^{\prime }$, H, $H^{\prime }$ such that


$$\begin{array}{c} N_{G}=N_{G^{\prime }}\text{ and }N_{H}=N_{H^{\prime }} \end{array}$$

whereas


$$\begin{array}{c} N_{G\circ H}\neq N_{G^{\prime }\circ H^{\prime }}. \end{array}$$

This fact implies, clearly, that $N_{G\circ H}$ cannot be computed directly from $N_{G}$ and $N_{H}$.

**Fig 9:**
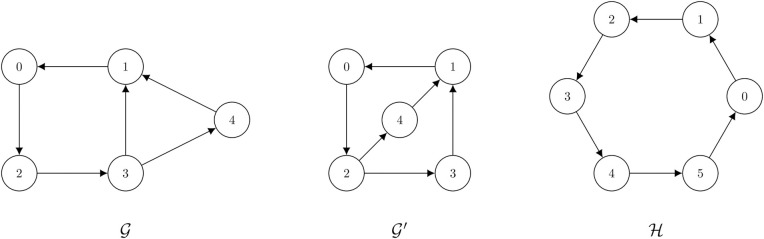
The graphs G and $G^{\prime }$ have the same DCM, but G∘H has a different DCM than $G^{\prime }\circ H$.

We choose the graphs G, $G^{\prime }$ and H as in Fig 9 (and let $H^{\prime }=H$). It is immediate to observe that


$$\begin{array}{c} N_{G}=N_{G^{\prime }}=(\begin{array}{ccccc} 1 & 1 & 2 & 1 & 0\\ 1 & 2 & 1 & 1 & 0\\ 1 & 1 & 1 & 2 & 0\\ 1 & 1 & 1 & 1 & 1\\ 1 & 1 & 1 & 1 & 1 \end{array}). \end{array}$$

To show that


$$\begin{array}{c} N_{G\circ H}\neq N_{G^{\prime }\circ H^{\prime }} \end{array}$$

we need the following:

**Definition 6.20 (Girth).** *For a graph* G *and a node* $x\in N_{G}$*, the local girth of* G *at x,* ${girth}_{G}(x)$*, is the length (number of arcs) of the shortest non-empty path from x to itself in* G *(*∞ *if such a path does not exist). Define*


$$\begin{array}{c} maxgirth(G)=\max_{x\in N_{G}}{girth}_{G}(x). \end{array}$$

Then we have:

**Theorem 6.21.** *Given two graphs* G *and* H *we have*


$$\begin{array}{c} diam(G\circ H)=\max(diam(G),\min(diam(H),maxgirth(G))). \end{array}$$

*Proof.* If $x,x^{\prime }\in N_{G}$ are two nodes realizing diam(G), then for any $y\in N_{H}$, the distance from (*x*,*y*) to $\left(x^{\prime },y\right)$ in G∘H is exactly diam(G). On the other hand, for any $x\in N_{G}$ and $y,y^{\prime }\in N_{H}$ we can build in G∘H a path from (*x*,*y*) to $\left(x,y^{\prime }\right)$ of length $d_{H}(y,y^{\prime })$ (by leaving the first coordinate fixed and moving the second coordinate along the path from *y* to $y^{\prime }$ in H), and another path of length ${girth}_{G}(x)$ (by moving along the non-empty path from *x* to *x* in G, while jumping from *y* to $y^{\prime }$ in H at any point along the path). □

As a consequence:

**Corollary 6.22.** *The lexicographic product is not DCM-stable on directed graphs.*

*Proof.* We must prove that $N_{G\circ H}\neq N_{G^{\prime }\circ H}$, for the graphs in Fig 9. We observe that $diam(G)=diam(G^{\prime })=4$, maxgirth(G)=5, $maxgirth(G^{\prime })=4$, diam(H)=5. Hence:


$$\begin{array}{c} diam(G\circ H)=\max(4,\min(5,5))=5 \end{array}$$

$$\begin{array}{c} diam(G^{\prime }\circ H)=\max(4,\min(5,4))=4. \end{array}$$

Two graphs with different finite diameters must have different DCMs. □

#### 6.2.4 Kronecker product.

**Fig 10:**
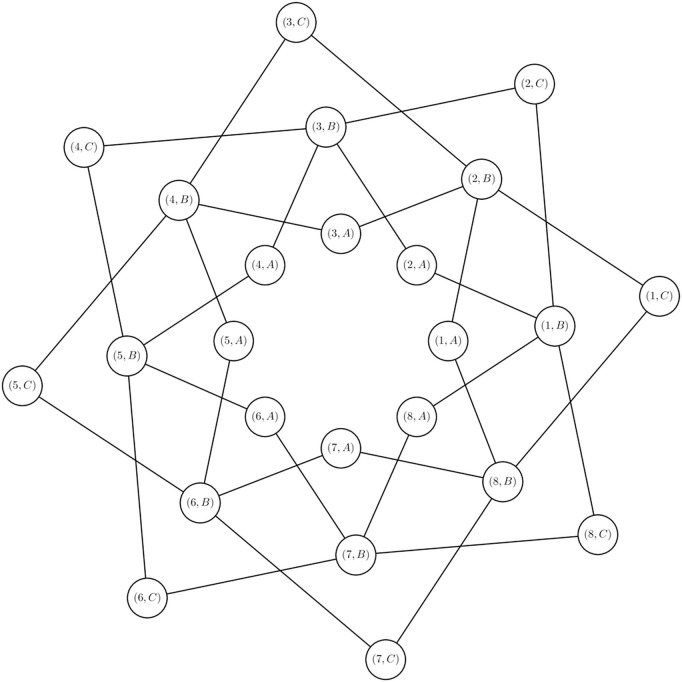
The Kronecker product G×H, where G is an undirected 8-cycle and H is an undirected 3-path (see Fig 5).

Not all natural graph operations (in particular: not all graph products) are DCM-stable. One example [13] is the following (see Fig 10):

**Definition 6.23 (Kronecker (or tensor) product).** *Given graphs* G *and* H*, their Kronecker product* G×H *has node set* $N_{G}\times N_{H}$ *and contains an arc* $\left((x,y),(x^{\prime },y^{\prime })\right)$ *if and only if* $(x,x^{\prime })\in E_{G}$ *and* $(y,y^{\prime })\in E_{H}$*.*

Note that a path in G×H can always be obtained by travelling in parallel along two paths of the same length (one in G and one in H). Even if either can be non-simple, the result might still be simple and in fact even a shortest path.

**Fig 11:**
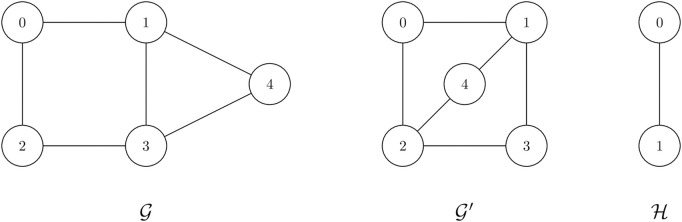
The graphs G, $G^{\prime }$ and H used to prove that the Kronecker product is not DCM-stable.

As we did for the lexicographic product, we provide an undirected counterexample: consider G, $G^{\prime }$ and H defined in Fig 11. We have that, up to node permutation,


$$\begin{array}{c} N_{G}=N_{G^{\prime }}=(\begin{array}{ccccc} 1 & 2 & 2 & 0 & 0\\ 1 & 2 & 2 & 0 & 0\\ 1 & 2 & 2 & 0 & 0\\ 1 & 3 & 1 & 0 & 0\\ 1 & 3 & 1 & 0 & 0 \end{array}). \end{array}$$

Nonetheless, from Theorem 1 of [31] we know that the Kronecker product of two connected graphs is connected if and only if one of them contains an odd cycle. In our example, only G contains an odd cycle. This implies that the graph G×H is connected, while $G^{\prime }\times H$ is not, which means that $N_{G\times H}\neq N_{G^{\prime }\times H}$.

**Corollary 6.24.** *Kronecker product is not DCM-stable. It is not even DCM-stable for undirected graphs.*

### 6.3 Corona product

The so-called corona product was originally defined by Harary and Frucht [32] for undirected graphs; here, we are going to borrow the three extensions of this product for the directed case proposed in [33], called *forward*, *backward*, and *symmetric*.

**Definition 6.25 (Corona product).** *Given graphs* G *and* H*, their forward* corona product $G\overset{\to }{⊙}H$*, backward* corona product $G\overset{\leftarrow }{⊙}H$*, and symmetric corona product* $G\overset{↔}{⊙}H$ *are the graphs having node set* $N_{G}\cup N_{G}\times N_{H}$ *and such that:*

- *all the arcs of* G *also belong to all the corona products;*
- *for every* $x\in N_{G}$ and any $(y,y^{\prime })\in E_{H}$, *there is an arc from* (*x*,*y*) to $\left(x,y^{\prime }\right)$ *in all the corona products;*
- *for every* $x\in N_{G}$ *and* $y\in N_{H}$
  – *there is an arc from x to* (*x*,*y*) *in* $G\overset{\to }{⊙}H$;
  – *there is an arc from* (*x*,*y*) *to x in* $G\overset{\leftarrow }{⊙}H$;
  – *there are two symmetric arcs connecting x with* (*x*,*y*) *in* $G\overset{↔}{⊙}H$.

**Fig 12:**
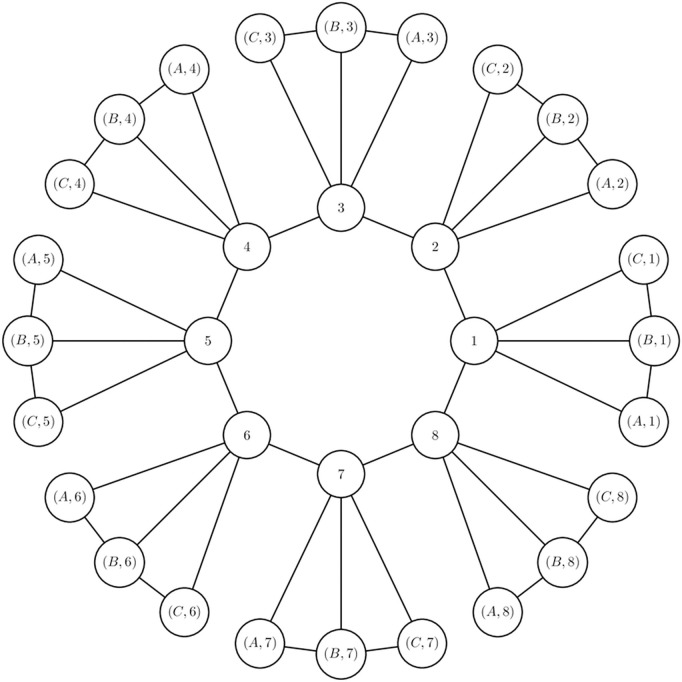
The corona product G⊙H, where G is an undirected 8-cycle and H is an undirected 3-path (see Fig 5). In the directed versions, the edges connecting G to the copies of H are directed outwards in $G\overset{\to }{⊙}H$, inwards in $G\overset{\leftarrow }{⊙}H$.

Alternatively, we can say that K contains one copy of G (the so-called “center graph”) and one copy of H for each node of G, and the three products differ only in the direction of the arcs between each node x∈G and the nodes of the copy of H associated with it. Note that $G\overset{↔}{⊙}H$ yields the same result as the original definition of the product if G and H are undirected. In such a case, we can refer to this operation simply as G⊙H (refer to Fig 12 for an example).

**Proposition 6.26.** *Let* G *and* H *be two graphs, and let* $K_{\to }=G\overset{\to }{⊙}H$*,* $K_{\leftarrow }=G\overset{\leftarrow }{⊙}H$*, and* $K_{↔}=G\overset{↔}{⊙}H$*. Then, for every* $x,x^{\prime }\in N_{G}$ *and* $y,y^{\prime }\in N_{H}$*, the distances between nodes in* $K_{\leftarrow }$*,* $K_{\to }$ *and* $K_{↔}$ *are shown in* Table 2*.*

**Table 2:** The distance between two nodes in the different versions of corona product.

*d*	$K_{\to }$	$K_{\leftarrow }$	$K_{↔}$
$x,x^{\prime }$	$d_{G}(x,x^{\prime })$	$d_{G}(x,x^{\prime })$	$d_{G}(x,x^{\prime })$
$x,(x^{\prime },y)$	$d_{G}(x,x^{\prime })+1$	∞	$d_{G}(x,x^{\prime })+1$
$(x^{\prime },y),x$	∞	$d_{G}(x^{\prime },x)+1$	$d_{G}(x^{\prime },x)+1$
$\left(x,y),(x^{\prime },y^{\prime }\right)$	$\left\{ \begin{array}{cc} d_{H}(y,y^{\prime }) & \text{if }x=x^{\prime }\\ \infty & \text{otherwise} \end{array}\right.$	$\left\{ \begin{array}{cc} d_{H}(y,y^{\prime }) & \text{if }x=x^{\prime }\\ \infty & \text{otherwise} \end{array}\right.$	$\left\{ \begin{array}{cc} \min(d_{H}(y,y^{\prime }),2) & \text{if }x=x^{\prime }\\ d_{G}(x,x^{\prime })+2 & \text{otherwise} \end{array}\right.$

As a consequence:

**Theorem 6.27.** *Let* G *and* H *be two graphs, and let* $K_{\to }=G\overset{\to }{⊙}H$*,* $K_{\leftarrow }=G\overset{\leftarrow }{⊙}H$*, and* $K_{↔}=G\overset{↔}{⊙}H$*. Then, for every* $x\in N_{G}$ *and* $y\in N_{H}$*:*

- $n_{K_{\to },k}(x)=n_{G,k}(x)$ and $n_{K_{\to },k}((x,y))=n_{H,k}(y)+n_{G,k-1}(x)$;
- $n_{K_{\leftarrow },k}(x)=n_{G,k}(x)+n_{G,k-1}(x)|N_{H}|$ and $n_{K_{\leftarrow },k}((x,y))=n_{H,k}(y)$;
- $n_{K_{↔},k}(x)=n_{G,k}(x)+n_{G,k-1}(x)|N_{H}|$ and

$$\begin{array}{c} n_{K_{↔},k}((x,y))=\left\{ \begin{array}{cc} 1 & \text{if }k=0\\ n_{H,1}(y)+1 & \text{if }k=1\\ n_{G,1}(x)+|N_{H}|-n_{H,1}(y)-1 & \text{if }k=2\\ n_{G,k-1}(x)+n_{G,k-2}(x)|N_{H}| & \text{if }k>2. \end{array}\right. \end{array}$$

*Proof.* Let us start with $x\in N_{G}$. Since it belongs to the center graph, regardless of the type of corona product we are considering, at a given distance *k* it has at least the same number of nodes it has at distance *k* in G. Additionally, for each $x^{\prime }\in N_{G}$ at distance k−1 in G, in $K_{\leftarrow }$ and $K_{↔}$ node *x* has at distance *k* the $\left|N_{H}\right|$ nodes belonging to the copy of H associated with (and, thus, connected to) $x^{\prime }$. Note that when *k* = 0, $n_{G,k-1}(x)=0$, thus $n_{K_{\leftarrow },k}(x)=n_{K_{↔},k}(x)=n_{G,k}(x)=1$, and when *k* = 1 the $\left|N_{H}\right|$ nodes at distance 1 to *x* in $K_{\leftarrow }$ and $K_{↔}$ are those belonging to the copy of H associated to *x* itself. Hence, in both cases, everything holds.

Moving to node (*x*,*y*), it is easy to see that in $K_{\to }$ and $K_{\leftarrow }$ it has at least the same number of nodes that *y* has at distance *k* in H. Additionally, in $K_{\to }$ in *k* steps (*x*,*y*) can also be reached by those nodes that can reach *x* in k−1 steps in G. In $K_{↔}$, instead, there are several cases to consider, based on the value of *k* (where *k* = 0 trivially holds). If *k* = 1, the nodes whose distance to (*x*,*y*) is 1 are the in-neighbors of *y* in H, plus *x*. When *k* = 2, instead, the nodes that can reach (*x*,*y*) in 2 steps are the in-neighbors of *x* in G, plus the $\left|N_{H}\right|$ nodes of the copy of H associated with *x*, from which we have to remove the in-neighbors of *y* and *y* itself. Finally, when *k* > 2, the nodes whose distance to (*x*,*y*) is *k* are those at distance k−1 to *x* in G, plus those belonging to copies of H associated with the nodes $x^{\prime }\in G$ such that $d_{G}(x^{\prime },x)=k-2$. □

**Corollary 6.28.** *The forward* $\overset{\to }{⊙}$*, backward* $\overset{\leftarrow }{⊙}$*, and symmetric* $\overset{↔}{⊙}$ *corona products of graphs are DCM-stable.*

## 7 Conclusions and future work

As mentioned, our negative result is especially relevant in the area of axiomatic centrality, when one tries to build counterexamples for specific properties of geometric centralities. On the other hand, there are families of graphs for which the DCM decision problem is feasible (e.g., out-directed trees), and we have shown that many natural and well-known graph operations can be expressed as operations between the corresponding distance-count matrices. Table 3 summarizes the results we obtained for the operations we considered. Our exploration in this direction is, at the moment, just a *ballon d’essai*, but it paves the way to pursue the problem of exploring the algebraic structure of DCMs to obtain positive and negative results on geometric centralities.

**Table 3:** DCM-stability of the graph operations discussed in this paper.

Operation	Undirected	Directed
Disjoint union	yes	
Strong product	yes	
Cartesian product	yes	
Lexicographic product	yes	no
Kronecker product	no	
Corona product	yes	

One problem that remains open is whether Theorem 5.5 can be extended to the case of general (not necessarily undirected) graphs. In particular, as we said, there are TPP instances **a** such that the matrix M(𝐚) is the DCM of some directed graph, even if **a** is a negative instance of Problem 5.2. We know that, if this happens, it must be a non-symmetric graph, because the symmetric case is already ruled out by our proof. We believe that also in the general case the problem remains NP-complete, but a formal proof of this fact is still missing.
