# Supplementary material for: Recognizing distance-count matrices
Source: PLoS One. 2026 Jul 8;21(7):e0352427. doi: 10.1371/journal.pone.0352427 (PMC13345339; doi:10.1371/journal.pone.0352427)
Supplement: Attachment — Submitted filename: reviews.pdf [file pone.0352427.s001.pdf]

Response to Reviewers

Reviewer #1

In “Recognizing Distance-Count Matrices”, the authors investigate so-called Distance Count Matrices (DCMs) associated to directed and undirected graphs. Such a matrix has at position (i, k) the number of nodes that sit at distance k to the node i, and thus (directly or indirectly) encodes many centrality properties of the graph.

In the first part of their paper, they show that deciding whether a given matrix is the DCM of an undirected graph is strongly NP-complete. This is a highly relevant result to the study of centrality, as it shows that it is not feasible to construct counterexamples to conjectures about DCMs, by first building a matrix with(out) required properties, and by then checking whether this matrix is the DCM of a graph. I believe this result about NP-completeness to have mathematical value in its own right, too.

In the second part of their work, they offer an alternative way of building DCMs, by showing that a great number of network products have DCMs that can be constructed from the DCMs of the constituents. By giving counter-examples, they prove that this is not the case for certain other products.

In my opinion, the paper is of very high quality. From what I can tell, there are (almost) no mistakes, the text is very complete and reads nicely, and above all it is highly original. Especially the idea for the proof of NP-completeness is very original, and frankly fun to read. I would only like to see most of the comments below addressed, but then I think it should be published.

Main

1. Subsection 5.1 was at times very unclear to me. Definition 5.1 defines a TPP to be a sequence of numbers with certain properties, but then Theorem 5.3 introduces additional properties to these sequences. Often times throughout the text it is not clear if what is being referred to is sequences with some or with all of these properties. The proof of Theorem 5.3, at the start, just refers to “the problem” for instance.

   > **A:** The reviewer is totally right. We now give a suitable name to the instances satisfying the additional properties needed by the NP-completeness result.

2. In subsection 6.4, it’s not always clear to me if we are considering directed or undirected graphs, especially around Proposition 6.30. Much more importantly though, I believe Proposition 6.30 to be wrong regardless. Consider the undirected chain graph with 8 nodes, $G = P_8$, consisting of the nodes 1 to 8, and the undirected 4-cycle, looking like A-B-C-D-, $H = C_4$. A surjective graph homomorphism is given by

   $$1 \to A,\ 2 \to B,\ 3 \to C,\ 4 \to D,\ 5 \to A,\ 6 \to B,\ 7 \to C,\ 8 \to D,$$

   I believe.

   Now, $m_{G,2}(1) = 3 < m_{H,2}(1) = 4$, if I am not mistaken. At any rate, I could not follow the proof of Proposition 6.30 very well. I believe the problem is that the restriction of the homomorphism from $M_{G,k}$ to $M_{H,k}$ may not be surjective itself, as the above counterexample shows. Clearly this part of the article is not the most important though.

   > **A:** The reviewer is right and the statement itself would need to be fixed. We decided to remove Subsection 6.4 altogether, because it does not add much and it is only partially

aligned with the rest of the paper.

Minor

1. The authors might consider giving a very basic example of network centrality already in the introduction. The reader less familiar with them now starts off somewhat lost, and has to wait to later sections to catch up.

    A: We now introduce very early harmonic centrality as an example of (linear) centrality, and explain how to express it in terms of the DCM.

2. On page 1, "and called its distance-count matrix (DCM)" → "and is called its distance-count matrix (DCM)"

    A: Rephrased.

3. On page 4, the paragraph starting with "Observe that" seems to be the only place where the subscript G is dropped from $M_{G,k}(x)$ and $N_{G,k}(x)$. I think this may confuse the reader.

    A: Fixed.

4. On page 4, "one special instance of linear centralities are…", these are even strictly linear, right?

    A: Correct; now we note it in parenthesis.

5. On page 5, the degree of P^i_g is at most $|N_G| - 1$, isn't it? See also Thr. 6.14 (though no conclusions are wrong).

    A: Fixed (both).

6. On page 5, "the standard reference is". I would put this more neutral; it may not be anymore in 10 years.

    A: Rephrased.

7. Proposition 4.7, I would briefly remind the reader that $n = \#G$

    A: Fixed.

8. Proposition 4.7,(i). Here $m_p(n-1)$ should probably be $m_{n-1}(i)$, correct?

    A: Sure: fixed.

9. The authors are not always very clear on if directed graphs can have self loops in this work or not. It seems mostly irrelevant for distance count issues, but Theorem 4.6 seems to me to only be true if self loops are allowed (because we allow $d_1 = p$), but Theorem 4.13 seems to only work (precisely as formulated) when we do not allow self loops. I think it's best to communicate to the reader what the convention will be early on.

    A: We fixed the statement and now assume explicitly that there are never self loops.

10. On page 8, in the inequality under "Therefore", the j_k should probably just be a j, right?

    A: Fixed.

11. On page 9, proof of Theorem 5.4, "the partition" should technically be "a partition", right? I see no reason to assume the solution, if it exists, is unique.

A: Fixed.

12. On page 10, "Finally, x_i has two vertices at distance three" $\to$ "four" (?)

A: Fixed.

13. On page 11, "$a_h + 1 \leq a_k + 2$, that is $a_h - a_k \leq 2$" $\to$ "that is $a_h - a_k \leq 1$"

A: Fixed.

14. In figure 6, aren't many arrows missing? Like from $(4, B)$ to $(3, C)$?

A: Fixed.

15. On page 15, the equation under "Recalling the definition of exponential-decay centrality, we have that", $((x, y)) \to (x, y)$ (too many brackets)

A: Fixed.

16. Lemma 6.17, "Propositon 5.12 of..." $\to$ "Proposition"

A: Fixed.

17. In Theorem 6.18, in the case where $n_{G,1}(x) = 0$, why would $n((x, y))$ be given by $n_{G,k}(x)|N_H|+ n_{H,k}(y)$ for $k > 0$, and not just by $n_{H,k}(y)$? After all, isn't it so that $n_{G,1}(x) = 0$ implies $n_{G,k}(x) = 0$ for all $k > 0$? Obviously nothing is wrong, but it might confuse the reader. After all, the fact that $n_{G,k}(x) = 0$ for all $k > 0$, also doesn't seem to be noticed in the proof.

A: Sure. Fixed.

18. On page 19, "the Kronecker product of two graphs is connected if and only if..." this should be the Kronecker product of two connected graphs, I think.

A: Fixed.

19. On page 22, in the proof of theorem 6.27, in the second line of the paragraph starting with "Moving to node (x, y)..." we have "Additionally, in $K_{\leftarrow}$ in k steps...", I believe the arrow has to be reversed here.

A: Fixed.

20. On page 23, the line above Proposition 6.30, the $n_{H,3}$ and $n_{G,3}$ should have an argument x, (it could be stated "for some x".)

A: Fixed.

21. On page 23, line 607, I believe $n_{G,1}(3) = 1$, not 0.

A: Fixed.

Reviewer #2

* Results are clear, but dependent on previously proved results, in new interpretations. Especially the results on distance degree sequences. For graph products also many results exist, the authors have proved them in different ways. Instead the authors are recommended to refer to the literature on graph products and distance degree sequences, to avoid repetitions.

A: In most cases, we just (re-)state them with appropriate reference and without proofs.

Some references that should have been checked first:

A: We added those references as previous results on distance degree sequences in products.

1. "Products of Distance Degree Regular and Distance Degree Injective graphs", Medha Itagi Huilgol, Rajeshwari M., Syed AsifUlla S., *Journal of Discrete Mathematical Sciences and Cryptography*, Vol. 15 (2012), No. 4&5, 303–314.

2. "New results on distance degree sequences of graphs", Medha Itagi Huilgol, V. Sriram, *Malaya Journal of Mathematik*, 7(2) (2019), 345–352.

3. "Tensor and Cartesian products for nanotori, nanotubes and zigzag polyhex nanotubes and their applications to ^{13}C NMR Spectroscopy", Medha Itagi Huilgol, V. Sriram, K. Balasubramanian, *Molecular Physics*, 118(16) (2020), 1–25. DOI: 00.1080/00268976.1817594

4. "Distance degree vector and scalar sequences of corona and lexicographic products of graphs with applications to dynamic NMR and dynamics of nonrigid molecules and proteins", Medha Itagi Huilgol, Divya B., K. Balasubramanian, *Theoretical Chemistry Accounts*, 140, 25 (2021). `https://doi.org/10.1007/s00214-021-02719-y`
