# Supplementary material for: Recognizing distance-count matrices
Source: PLoS One. 2026 Jul 8;21(7):e0352427. doi: 10.1371/journal.pone.0352427 (PMC13345339; doi:10.1371/journal.pone.0352427)
Supplement: Peer review history

# Peer review: Decision Letter 0

**DOI:** 10.1371/journal.pone.0352427.r001

**Authors:** Tiago Pereira

20 Apr 2026

PONE-D-26-09672

Recognizing Distance-Count Matrices

PLOS One

Dear Dr. BOLDI,

Thank you for submitting your manuscript to PLOS ONE. After careful consideration, we feel that it has merit but does not fully meet PLOS ONE’s publication criteria as it currently stands. Therefore, we invite you to submit a revised version of the manuscript that addresses the points raised during the review process.

Please submit your revised manuscript by Jun 04 2026 11:59PM. If you will need more time than this to complete your revisions, please reply to this message or contact the journal office at plosone@plos.org. When you're ready to submit your revision, log on to https://www.editorialmanager.com/pone/ and select the 'Submissions Needing Revision' folder to locate your manuscript file.

Please include the following items when submitting your revised manuscript:

- A letter that responds to each point raised by the academic editor and reviewer(s). You should upload this letter as a separate file labeled 'Response to Reviewers'.
- A marked-up copy of your manuscript that highlights changes made to the original version. You should upload this as a separate file labeled 'Revised Manuscript with Track Changes'.
- An unmarked version of your revised paper without tracked changes. You should upload this as a separate file labeled 'Manuscript'.

If you would like to make changes to your financial disclosure, please include your updated statement in your cover letter. Guidelines for resubmitting your figure files are available below the reviewer comments at the end of this letter.

If applicable, we recommend that you deposit your laboratory protocols in protocols.io to enhance the reproducibility of your results. Protocols.io assigns your protocol its own identifier (DOI) so that it can be cited independently in the future. For instructions see: https://journals.plos.org/plosone/s/submission-guidelines#loc-laboratory-protocols. Additionally, PLOS ONE offers an option for publishing peer-reviewed Lab Protocol articles, which describe protocols hosted on protocols.io. Read more information on sharing protocols at https://plos.org/protocols?utm_medium=editorial-email&utm_source=authorletters&utm_campaign=protocols.

As the corresponding author, your ORCID iD is verified in the submission system and will appear in the published article. PLOS supports the use of ORCID, and we encourage all coauthors to register for an ORCID iD and use it as well. Please encourage your coauthors to verify their ORCID iD within the submission system before final acceptance, as unverified ORCID iDs will not appear in the published article. *Only*  the individual author can complete the verification step; PLOS staff *cannot*  verify ORCID iDs on behalf of authors.

We look forward to receiving your revised manuscript.

Kind regards,

Tiago Pereira

Academic Editor

PLOS One

**Journal Requirements:**

When submitting your revision, we need you to address these additional requirements.

1. Please ensure that your manuscript meets PLOS ONE's style requirements, including those for file naming. The PLOS ONE style templates can be found at

https://journals.plos.org/plosone/s/file?id=wjVg/PLOSOne_formatting_sample_main_body.pdf and

https://journals.plos.org/plosone/s/file?id=ba62/PLOSOne_formatting_sample_title_authors_affiliations.pdf

2. Please note that PLOS One has specific guidelines on code sharing for submissions in which author-generated code underpins the findings in the manuscript. In these cases, we expect all author-generated code to be made available without restrictions upon publication of the work. Please review our guidelines at https://journals.plos.org/plosone/s/materials-and-software-sharing#loc-sharing-code and ensure that your code is shared in a way that follows best practice and facilitates reproducibility and reuse.

3. Please update your submission to use the PLOS LaTeX template. The template and more information on our requirements for LaTeX submissions can be found at http://journals.plos.org/plosone/s/latex.

4. Please ensure that you refer to Figures 2, 5, 6, 7, 8, and 10 in your text as, if accepted, production will need this reference to link the reader to the figure.

5. If the reviewer comments include a recommendation to cite specific previously published works, please review and evaluate these publications to determine whether they are relevant and should be cited. There is no requirement to cite these works unless the editor has indicated otherwise.

6. Please review your reference list to ensure that it is complete and correct. If you have cited papers that have been retracted, please include the rationale for doing so in the manuscript text, or remove these references and replace them with relevant current references. Any changes to the reference list should be mentioned in the rebuttal letter that accompanies your revised manuscript. If you need to cite a retracted article, indicate the article’s retracted status in the References list and also include a citation and full reference for the retraction notice.

[Note: HTML markup is below. Please do not edit.]

Reviewers' comments:

Reviewer's Responses to Questions

**Comments to the Author**

1. Is the manuscript technically sound, and do the data support the conclusions?

The manuscript must describe a technically sound piece of scientific research with data that supports the conclusions. Experiments must have been conducted rigorously, with appropriate controls, replication, and sample sizes. The conclusions must be drawn appropriately based on the data presented.

Reviewer #1: Yes

Reviewer #2: Yes

**********

2. Has the statistical analysis been performed appropriately and rigorously?

Reviewer #1: Yes

Reviewer #2: Yes

**********

3. Have the authors made all data underlying the findings in their manuscript fully available?

The PLOS Data policy requires authors to make all data underlying the findings described in their manuscript fully available without restriction, with rare exception (please refer to the Data Availability Statement in the manuscript PDF file). The data should be provided as part of the manuscript or its supporting information, or deposited to a public repository. For example, in addition to summary statistics, the data points behind means, medians and variance measures should be available. If there are restrictions on publicly sharing data—e.g. participant privacy or use of data from a third party—those must be specified.

Reviewer #1: Yes

Reviewer #2: Yes

**********

4. Is the manuscript presented in an intelligible fashion and written in standard English?

PLOS ONE does not copyedit accepted manuscripts, so the language in submitted articles must be clear, correct, and unambiguous. Any typographical or grammatical errors should be corrected at revision, so please note any specific errors here.

Reviewer #1: Yes

Reviewer #2: Yes

**********

5. Review Comments to the Author

Please use the space provided to explain your answers to the questions above. You may also include additional comments for the author, including concerns about dual publication, research ethics, or publication ethics. (Please upload your review as an attachment if it exceeds 20,000 characters)

Reviewer #1: In "Recognizing Distance-Count Matrices", the authors investigate so-called Distance Count Matrices (DCMs) associated to directed and undirected graphs. Such a matrix has at position (i,k) the number of nodes that sit at distance k to the node i, and thus (directly or indirectly) encodes many centrality properties of the graph.

In the first part of their paper, they show that deciding whether a given matrix is the DCM of an undirected graph is strongly NP-complete. This is a highly relevant result to the study of centrality, as it shows that it is not feasible to construct counterexamples to conjectures about DCMs, by first building a matrix with(out) required properties, and by then checking whether this matrix is the DCM of a graph. I believe this result about NP-completeness to have mathematical value in its own right, too.

In the second part of their work, they offer an alternative way of building DCMs, by showing that a great number of network products have DCMs that can be constructed from the DCMs of the constituents. By giving counter-examples, they prove that this is not the case for certain other products.

In my opinion, the paper is of very high quality. From what I can tell, there are (almost) no mistakes, the text is very complete and reads nicely, and above all it is highly original. Especially the idea for the proof of NP-completeness is very original, and frankly fun to read. I would only like to see most of the comments below addressed, but then I think it should be published.

Main:

1) Subsection 5.1 was at times very unclear to me. Definition 5.1 defines a TPP to be a sequence of numbers with certain properties, but then Theorem 5.3 introduces additional properties to these sequences. Often times throughout the text it is not clear if what is being referred to is sequences with some or with all of these properties. The proof of Theorem 5.3, at the start, just refers to "the problem" for instance.

2) In subsection 6.4, it's not always clear to me if we are considering directed or undirected graphs, especially around Proposition 6.30. Much more importantly though, I believe Proposition 6.30 to be wrong regardless. Consider the undirected chain graph with 8 nodes, G = P_8, consisting of the nodes 1 to 8, and the undirected 4-cycle, looking like A-B-C-D-, H = C4. A surjective graph homomorphism is given by

1->A, 2->B, 3->C, 4->D, 5->A, 6->B, 7->C, 8->D,

I believe.

Now, m_{G,2}(1) = 3 < m_{H,2}(1) = 4, if I am not mistaken. At any rate, I could not follow the proof of Proposition 6.30 very well. I believe the problem is that the restriction of the homomorphism from M_{G,k} to M_{H,k} may not be surjective itself, as the above counterexample shows. Clearly this part of the article is not the most important though.

Minor:

1) The authors might consider giving a very basic example of network centrality already in the introduction. The reader less familiar with them now starts off somewhat lost, and has to wait to later sections to catch up.

2) On page 1, "and called its distance-count matrix (DCM)" -> " and is called its distance-count matrix (DCM)"

3) On page 4, the paragraph starting with "Observe that" seems to be the only place where the subscript G is dropped from M_{G,k}(x) and N_{G,k}(x). I think this may confuse the reader.

4) On page 4, "one special instance of linear centralities are...", these are even strictly linear, right?

5) On page 5, the degree of P_g^i is at most |N_G|-1, isn't it? See also Thr. 6.14 (though no conclusions are wrong).

6) On page 5, "the standard reference is". I would put this more neutral; it may not be anymore in 10 years.

7) Proposition 4.7, I would briefly remind the reader that n = #G

8) Proposition 4.7,(i). Here m_{p}(n-1) should probably be m_{n-1}(i), correct?

9) The authors are not always very clear on if directed graphs can have self loops in this work or not. It seems mostly irrelevant for distance count issues, but Theorem 4.6 seems to me to only be true if self loops are allowed (because we allow d_1 = p), but Theorem 4.13 seems to only work (precisely as formulated) when we do not allow self loops. I think it's best to communicate to the reader what the convention will be early on.

10) On page 8, in the inequality under "Therefore", the j_k should probably just be a j, right?

11) On page 9, proof of Theorem 5.4, "the partition" should technically be "a partition", right? I see no reason to assume the solution, if it exists, is unique.

12) On page 10, "Finally, x_i has two vertices at distance three" --> "four" (?)

13) On page 11, "a_h + 1 \leq a_k + 2, that is a_h - a_k \leq 2" --> "that is a_h - a_k \leq 1"

14) In figure 6, aren't many arrows missing? Like from (4,B) to (3,C)?

15) On page 15, the Equation under "Recalling the definition of exponential-decay centrality, we have that", ((x,y)) ---> (x,y) (too many brackets)

16) Lemma 6.17, "Propositon 5.12 of ..." --> "Proposition"

17) In Theorem 6.18, in the case where n_{G,1}(x) = 0, why would n((x,y)) be given by n_{G,k}(x)|N_H| + n_{H,k}(y) for k>0, and not just by n_{H,k}(y)? after all, isn't it so that n_{G,1}(x) = 0 implies n_{G,k}(x) = 0 for all k>0? Obviously nothing is wrong, but it might confuse the reader. After all, the fact that n_{G,k}(x) = 0 for all k>0, also doesn't seem to be noticed in the proof.

18) On page 19, "the Kronecker product of two graphs is connected if and only if ...." this should be the Kronecker product of two connected graphs, I think.

19) On page 22, In the proof of theorem 6.27, in the second line of the paragraph starting with "Moving to node (x,y)..." we have "Additionally, in K_{left arrow} in k steps ... ", I believe the arrow has to be reversed here.

20) On page 23, the line above Proposition 6.30, the n_{H,3} and n_{G,3} should have an argument x, (it could be stated "for some x".)

21) On page 23, line 607, I believe n_{G,1}(3) = 1, not 0.

Reviewer #2: * Results are clear, but dependent on previously proved results, in new interpretations. Especially the results on distance degree sequences. For graph products also many results exist, the authors have proved them in different ways. Instead the authors are recommended to refer to the literature on graph products and distance degree sequences, to avoid repetitions. Some references that should have been checked first:

1. “Products of Distance Degree Regular and Distance Degree Injective graphs”, Medha Itagi Huilgol, Rajeshwari M., Syed AsifUlla S., Journal of Discrete Mathematical Sciences and Cryptography, Vol.15 (2012), No. 4&5, 303-314.

2. “New results on distance degree sequences of graphs”, Medha Itagi Huilgol, V. Sriram, Malaya Journal of Mathematik, 7(2) (2019), 345-352.

3. . “Tensor and Cartesian products for nanotori, nanotubes and zigzag polyhex nanotubes and their applications to 13C NMR Spectroscopy”, Medha Itagi Huilgol, V. Sriram, K. Balasubramanian, Molecular Physics, 118(16) (2020), 1-25.

DOI: 00.1080/00268976.1817594

4. “Distance degree vector and scalar sequences of corona and lexicographic products of graphs with applications to dynamic NMR and dynamics of nonrigid molecules and proteins”, Medha Itagi Huilgol, Divya B., K. Balasubramanian, Theoretical Chemistry Accounts, 140, 25 (2021). https://doi.org/10.1007/s00214-021-02719-y

* The authors are recommended to do a thorough literature survey and improve the results.

With corrections incorporated, the authors may resubmit.

**********

6. PLOS authors have the option to publish the peer review history of their article (what does this mean?). If published, this will include your full peer review and any attached files.

If you choose “no”, your identity will remain anonymous but your review may still be made public.

**Do you want your identity to be public for this peer review?**  For information about this choice, including consent withdrawal, please see our Privacy Policy.

Reviewer #1: No

Reviewer #2: No

**********

[NOTE: If reviewer comments were submitted as an attachment file, they will be attached to this email and accessible via the submission site. Please log into your account, locate the manuscript record, and check for the action link "View Attachments". If this link does not appear, there are no attachment files.]

To ensure your figures meet our technical requirements, please review our figure guidelines: https://journals.plos.org/plosone/s/figures

You may also use PLOS’s free figure tool, NAAS, to help you prepare publication quality figures: https://journals.plos.org/plosone/s/figures#loc-tools-for-figure-preparation.

NAAS will assess whether your figures meet our technical requirements by comparing each figure against our figure specifications.

# Author comment: Author response to Decision Letter 1

**DOI:** 10.1371/journal.pone.0352427.r002

25 May 2026

Reviewer #1: In "Recognizing Distance-Count Matrices", the authors investigate so-called Distance Count Matrices (DCMs) associated to directed and undirected graphs. Such a matrix has at position (i,k) the number of nodes that sit at

distance k to the node i, and thus (directly or indirectly) encodes many centrality properties of the graph.

In the first part of their paper, they show that deciding whether a given matrix is the DCM of an undirected graph is strongly NP-complete. This is a highly relevant result to the study of centrality, as it shows that it is not feasible to

construct counterexamples to conjectures about DCMs, by first building a matrix with(out) required properties, and by then checking whether this matrix is the DCM of a graph. I believe this result about NP-completeness to have mathematical

value in its own right, too.

In the second part of their work, they offer an alternative way of building DCMs, by showing that a great number of network products have DCMs that can be constructed from the DCMs of the constituents. By giving counter-examples, they prove

that this is not the case for certain other products.

In my opinion, the paper is of very high quality. From what I can tell, there are (almost) no mistakes, the text is very complete and reads nicely, and above all it is highly original. Especially the idea for the proof of NP-completeness is

very original, and frankly fun to read. I would only like to see most of the comments below addressed, but then I think it should be published.

Main:

1) Subsection 5.1 was at times very unclear to me. Definition 5.1 defines a TPP to be a sequence of numbers with certain properties, but then Theorem 5.3 introduces additional properties to these sequences. Often times throughout the text

it is not clear if what is being referred to is sequences with some or with all of these properties. The proof of Theorem 5.3, at the start, just refers to "the problem" for instance.

A: The reviewer is totally right. We now give a suitable name to the instances satisfying the additional properties needed by the NP-completeness result.

2) In subsection 6.4, it's not always clear to me if we are considering directed or undirected graphs, especially around Proposition 6.30. Much more importantly though, I believe Proposition 6.30 to be wrong regardless. Consider the

undirected chain graph with 8 nodes, G = P_8, consisting of the nodes 1 to 8, and the undirected 4-cycle, looking like A-B-C-D-, H = C4. A surjective graph homomorphism is given by

1->A, 2->B, 3->C, 4->D, 5->A, 6->B, 7->C, 8->D,

I believe.

Now, m_{G,2}(1) = 3 < m_{H,2}(1) = 4, if I am not mistaken. At any rate, I could not follow the proof of Proposition 6.30 very well. I believe the problem is that the restriction of the homomorphism from M_{G,k} to M_{H,k} may not be

surjective itself, as the above counterexample shows. Clearly this part of the article is not the most important though.

A: The reviewer is right and the statement itself would need to be fixed. We decided to remove Subsection 6.4 altogether, because it does not add much and it is only partially aligned with the rest of the paper.

Minor:

1) The authors might consider giving a very basic example of network centrality already in the introduction. The reader less familiar with them now starts off somewhat lost, and has to wait to later sections to catch up.

A: We now introduce very early harmonic centrality as an example of (linear) centrality, and explain how to express it in terms of the DCM.

2) On page 1, "and called its distance-count matrix (DCM)" -> " and is called its distance-count matrix (DCM)"

A: Rephrased.

3) On page 4, the paragraph starting with "Observe that" seems to be the only place where the subscript G is dropped from M_{G,k}(x) and N_{G,k}(x). I think this may confuse the reader.

A: Fixed.

4) On page 4, "one special instance of linear centralities are...", these are even strictly linear, right?

A: Correct; now we note it in parenthesis.

5) On page 5, the degree of P_g^i is at most |N_G|-1, isn't it? See also Thr. 6.14 (though no conclusions are wrong).

A: Fixed (both).

6) On page 5, "the standard reference is". I would put this more neutral; it may not be anymore in 10 years.

A: Rephrased.

7) Proposition 4.7, I would briefly remind the reader that n = #G

A: Fixed.

8) Proposition 4.7,(i). Here m_{p}(n-1) should probably be m_{n-1}(i), correct?

A: Sure: fixed.

9) The authors are not always very clear on if directed graphs can have self loops in this work or not. It seems mostly irrelevant for distance count issues, but Theorem 4.6 seems to me to only be true if self loops are allowed (because we

allow d_1 = p), but Theorem 4.13 seems to only work (precisely as formulated) when we do not allow self loops. I think it's best to communicate to the reader what the convention will be early on.

A: We fixed the statement and now assume explicitly that there

are never self loops.

10) On page 8, in the inequality under "Therefore", the j_k should probably just be a j, right?

A: Fixed.

11) On page 9, proof of Theorem 5.4, "the partition" should technically be "a partition", right? I see no reason to assume the solution, if it exists, is unique.

A: Fixed.

12) On page 10, "Finally, x_i has two vertices at distance three" --> "four" (?)

A: Fixed.

13) On page 11, "a_h + 1 \leq a_k + 2, that is a_h - a_k \leq 2" --> "that is a_h - a_k \leq 1"

A: Fixed.

14) In figure 6, aren't many arrows missing? Like from (4,B) to (3,C)?

A: Fixed.

15) On page 15, the Equation under "Recalling the definition of exponential-decay centrality, we have that", ((x,y)) ---> (x,y) (too many brackets)

A: Fixed.

16) Lemma 6.17, "Propositon 5.12 of ..." --> "Proposition"

A: Fixed.

17) In Theorem 6.18, in the case where n_{G,1}(x) = 0, why would n((x,y)) be given by n_{G,k}(x)|N_H| + n_{H,k}(y) for k>0, and not just by n_{H,k}(y)? after all, isn't it so that n_{G,1}(x) = 0 implies n_{G,k}(x) = 0 for all k>0? Obviously

nothing is wrong, but it might confuse the reader. After all, the fact that n_{G,k}(x) = 0 for all k>0, also doesn't seem to be noticed in the proof.

A: Sure. Fixed.

18) On page 19, "the Kronecker product of two graphs is connected if and only if ...." this should be the Kronecker product of two connected graphs, I think.

A: Fixed.

19) On page 22, In the proof of theorem 6.27, in the second line of the paragraph starting with "Moving to node (x,y)..." we have "Additionally, in K_{left arrow} in k steps ... ", I believe the arrow has to be reversed here.

A: Fixed.

20) On page 23, the line above Proposition 6.30, the n_{H,3} and n_{G,3} should have an argument x, (it could be stated "for some x".)

A: Fixed.

21) On page 23, line 607, I believe n_{G,1}(3) = 1, not 0.

A: Fixed.

---------

Reviewer #2: * Results are clear, but dependent on previously proved results, in new interpretations. Especially the results on distance degree sequences. For graph products also many results exist, the authors have proved them in different

ways. Instead the authors are recommended to refer to the literature on graph products and distance degree sequences, to avoid repetitions.

A: In most cases, we just (re-)state them with appropriate reference and without proofs.

Some references that should have been checked first:

A: We added those references as previous results on distance degree sequences in products.

1. “Products of Distance Degree Regular and Distance Degree Injective graphs”, Medha Itagi Huilgol, Rajeshwari M., Syed AsifUlla S., Journal of Discrete Mathematical Sciences and Cryptography, Vol.15 (2012), No. 4&5, 303-314.

2. “New results on distance degree sequences of graphs”, Medha Itagi Huilgol, V. Sriram, Malaya Journal of Mathematik, 7(2) (2019), 345-352.

3. . “Tensor and Cartesian products for nanotori, nanotubes and zigzag polyhex nanotubes and their applications to 13C NMR Spectroscopy”, Medha Itagi Huilgol, V. Sriram, K. Balasubramanian, Molecular Physics, 118(16) (2020), 1-25.

DOI: 00.1080/00268976.1817594

4. “Distance degree vector and scalar sequences of corona and lexicographic products of graphs with applications to dynamic NMR and dynamics of nonrigid molecules and proteins”, Medha Itagi Huilgol, Divya B., K. Balasubramanian, Theoretical

Chemistry Accounts, 140, 25 (2021). https://doi.org/10.1007/s00214-021-02719-y

Attachment Submitted filename: reviews.pdf

# Editor report: Decision Letter 1

**DOI:** 10.1371/journal.pone.0352427.r003

**Authors:** Tiago Pereira

11 Jun 2026

Recognizing Distance-Count Matrices

PONE-D-26-09672R1

Dear Dr. BOLDI,

We’re pleased to inform you that your manuscript has been judged scientifically suitable for publication and will be formally accepted for publication once it meets all outstanding technical requirements.

Within one week, you’ll receive an e-mail detailing the required amendments. When these have been addressed, you’ll receive a formal acceptance letter and your manuscript will be scheduled for publication.

An invoice will be generated when your article is formally accepted. Please note, if your institution has a publishing partnership with PLOS and your article meets the relevant criteria, all or part of your publication costs will be covered. Please make sure your user information is up-to-date by logging into Editorial Manager at Editorial Manager® and clicking the ‘Update My Information' link at the top of the page. For questions related to billing, please contact billing support.

If your institution or institutions have a press office, please notify them about your upcoming paper to help maximize its impact. If they’ll be preparing press materials, please inform our press team as soon as possible -- no later than 48 hours after receiving the formal acceptance. Your manuscript will remain under strict press embargo until 2 pm Eastern Time on the date of publication. For more information, please contact onepress@plos.org.

Kind regards,

Tiago Pereira

Academic Editor

PLOS One

Additional Editor Comments (optional):

Reviewers' comments:

# Editor report: Acceptance letter

**DOI:** 10.1371/journal.pone.0352427.r004

**Authors:** Tiago Pereira

PONE-D-26-09672R1

PLOS One

Dear Dr. BOLDI,

I'm pleased to inform you that your manuscript has been deemed suitable for publication in PLOS One. Congratulations! Your manuscript is now being handed over to our production team.

At this stage, our production department will prepare your paper for publication. This includes ensuring the following:

* All references, tables, and figures are properly cited

* All relevant supporting information is included in the manuscript submission,

* There are no issues that prevent the paper from being properly typeset

You will receive further instructions from the production team, including instructions on how to review your proof when it is ready. Please keep in mind that we are working through a large volume of accepted articles, so please give us a few days to review your paper and let you know the next and final steps.

Lastly, if your institution or institutions have a press office, please let them know about your upcoming paper now to help maximize its impact. If they'll be preparing press materials, please inform our press team within the next 48 hours. Your manuscript will remain under strict press embargo until 2 pm Eastern Time on the date of publication. For more information, please contact onepress@plos.org.

You will receive an invoice from PLOS for your publication fee after your manuscript has reached the completed accept phase. If you receive an email requesting payment before acceptance or for any other service, this may be a phishing scheme. Learn how to identify phishing emails and protect your accounts at https://explore.plos.org/phishing.

If we can help with anything else, please email us at customercare@plos.org.

Thank you for submitting your work to PLOS ONE and supporting open access.

Kind regards,

PLOS ONE Editorial Office Staff

on behalf of

Dr. Tiago Pereira

Academic Editor

PLOS One
